# Utilizing direct and indirect information to improve the COVID-19 vaccination booster scheduling

**DOI:** 10.1038/s41598-024-58690-8

**Published:** 2024-04-06

**Authors:** Yotam Dery, Matan Yechezkel, Irad Ben-Gal, Dan Yamin

**Affiliations:** 1 Laboratory for Epidemic Modeling and Analysis, Department of Industrial Engineering, Faculty of Engineering, Tel Aviv University, 6997801 Tel Aviv, Israel; 2 Laboratory for AI, Machine Learning and Business Data Analytics, Tel Aviv University, 6997801 Tel Aviv, Israel; 3 Centre for Combatting Pandemics, Tel Aviv University, 6997801 Tel Aviv, Israel

**Keywords:** Value of information, Vaccination, COVID-19, Transmission model, SEIR model, Health policy, Epidemiology

## Abstract

Current global COVID-19 booster scheduling strategies mainly focus on vaccinating high-risk populations at predetermined intervals. However, these strategies overlook key data: the direct insights into individual immunity levels from active serological testing and the indirect information available either through sample-based sero-surveillance, or vital demographic, location, and epidemiological factors. Our research, employing an age-, risk-, and region-structured mathematical model of disease transmission—based on COVID-19 incidence and vaccination data from Israel between 15 May 2020 and 25 October 2021—reveals that a more comprehensive strategy integrating these elements can significantly reduce COVID-19 hospitalizations without increasing existing booster coverage. Notably, the effective use of indirect information alone can considerably decrease COVID-19 cases and hospitalizations, without the need for additional vaccine doses. This approach may also be applicable in optimizing vaccination strategies for other infectious diseases, including influenza.

## Introduction

Common vaccination strategies for infectious diseases are often categorized as morbidity-based or mortality-based. These approaches prioritize subpopulations that are either most responsible for transmission or at the highest risk of complications. Despite the World Health Organization (WHO) declaring the end of the pandemic phase of severe acute respiratory syndrome coronavirus 2 (SARS-CoV-2), it remains a significant health threat, particularly for individuals over 60 and those with certain medical conditions^1,2^. Thus, current global COVID-19 booster scheduling strategies focus on vaccinating these high-risk groups at set intervals^3,4^. These strategies are largely mortality-based, as per clinical guidelines, targeting primarily the elderly and vulnerable populations first. Booster vaccines are initially offered to the most susceptible, such as the elderly (60+) and individuals with pre-existing conditions, before being gradually extended to children, younger adults, and the rest of the population^1,4–9,22^.

The effectiveness of COVID-19 booster vaccine doses diminishes over time due to natural immunity waning and the emergence of new SARS-CoV-2 variants. Studies suggest that a prior COVID-19 infection reduces the risk of subsequent severe infection for an average period of 9 months^10,11^, while a booster vaccine dose provides protection for up to 6 months only^12–14^. Furthermore, COVID-19 surges do not impact all regions uniformly, often resulting in local hotspots^15^. Various location-related factors, including demographics and socioeconomic status, influence the risk of COVID infection and its severity^15,16^. These findings underscore that the virus’s dynamics are highly time-dependent^17,18^. Therefore, leveraging local immunity levels is crucial in developing a vaccination strategy that prioritizes the most vulnerable individuals within the targeted population. Such a strategy has the potential to reduce COVID-19 hospitalizations and deaths more efficiently and effectively than previous methods.

Immunity levels can be indirectly inferred by analyzing location and temporal data. For instance, in subpopulations where recent higher transmission rates are observed, individuals are more likely to have temporary immunity. A more proactive, yet indirect method is the implementation of frequent sample-based sero-surveillance. This involves testing a small but representative sample from each subpopulation to estimate its overall immunity level. In this regard, while costly, plaque reduction neutralization tests can be integral to sample-based sero-surveillance. Their significance lies in guiding and justifying vaccine administration, especially considering their ability to align with observed trends^19^. Subject to their sensitivity and specificity in identifying protection against circulating variants, direct inference of immunity levels can also be achieved through individual rapid serology tests^20^. These tests can identify previous COVID-19 infections, including in asymptomatic individuals.

In this paper, we demonstrate the value of both direct and indirect information in understanding the dynamics and transmission of COVID-19, using Israel as a case study. We incorporated extensive data from anonymized health records provided by Israel's Ministry of Health into a regional, age-, and risk-structured transmission model. With just five key estimated parameters, our model shows that employing direct and indirect information can significantly reduce the number of COVID-19 infections and hospitalizations compared to current global policy strategies. Our methodology offers policymakers a way to leverage such information, enabling them to prioritize and develop more effective booster vaccination strategies.

## Results

### Dynamic model

We utilized publicly available, daily data on COVID-19 reported cases, tests, and vaccinations (1st, 2nd, and 3rd doses) from the Israeli Ministry of Health for the period from 15 May 2020 to 25 October 2021. Individuals were classified as having COVID-19 if they tested positive on an RT-PCR test. This dataset, encompassing the entire Israeli population, is stratified into 1642 statistical regions as defined by the Israeli Central Bureau of Statistics. We leveraged the demographic data of each statistical region and consolidated the 1642 regions into 30 larger regions.

During this period, the emergence of new variants and the rapid waning of antibodies contributed to infection surges. In response, Israeli policymakers implemented various restrictions to curb virus transmission. However, adherence to these provisions varied across the population. To quantify this variance, we used a workplace attendance parameter derived from data on changes in workplace visits since the pandemic's onset in Israel. The baseline for this parameter was set as the median number of workplace visits during the 5-week period from 3 January to 6 February 2020. This parameter correlates with reported case numbers in Israel during times of relaxed restrictions or high daily case rates.

**Figure 1:**
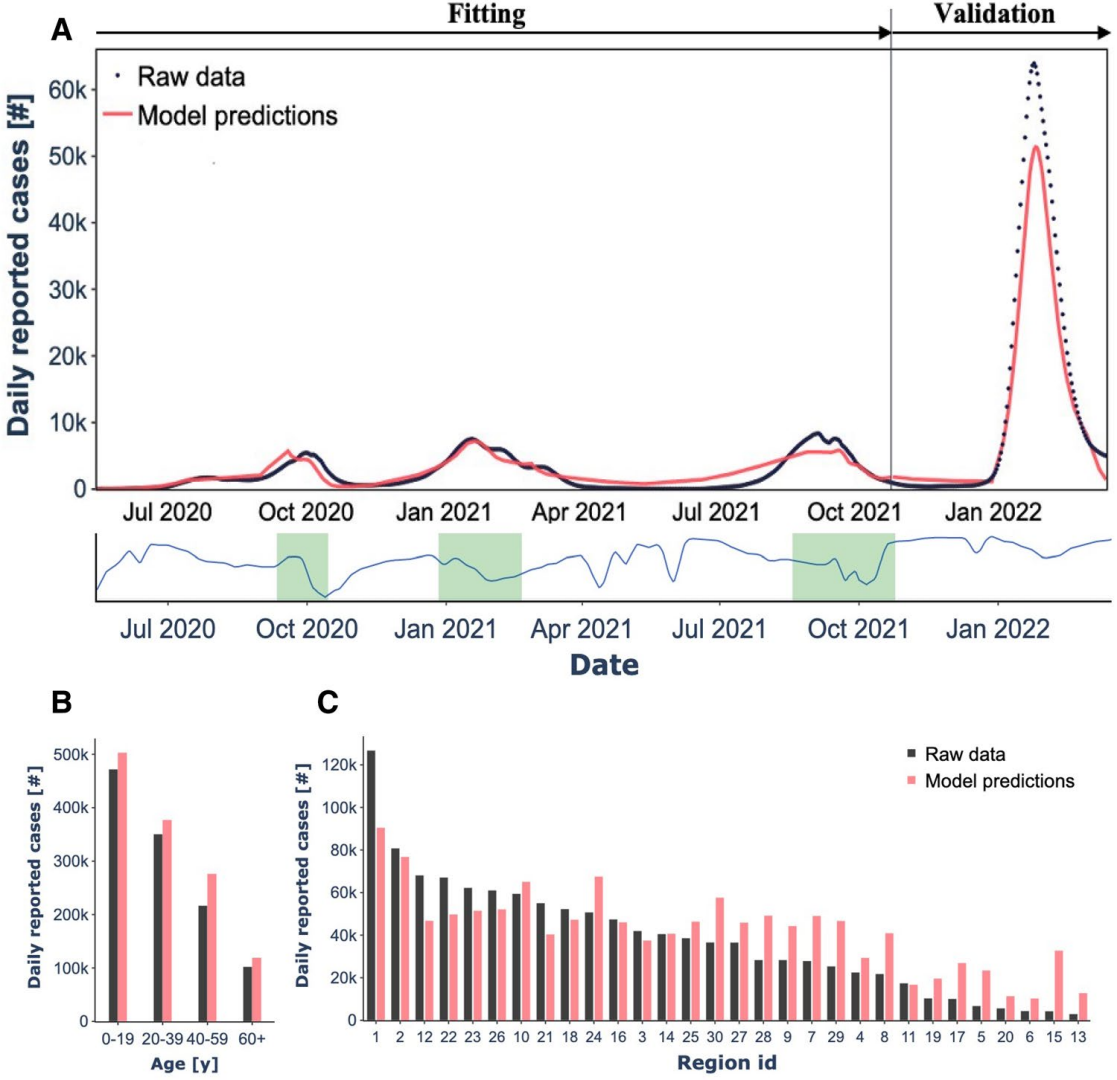
Fit of the transmission model under the scenario of three unreported cases per reported case. (**a**) Upper panel—Time series of the total number of daily reported cases of COVID-19 countrywide. We performed the model fit and validation utilizing the total number of daily reported cases. Lower panel—descriptive visualization of the time periods in which the ${\widehat{\beta }}_{workplace}$ influenced the transmission model (**b**). Data and model fit to the total number of reported COVID-19 cases among different age groups. (**c**) Data and model fit to the 30 regions covering Israel.

Our analysis incorporated this data into an age-, region-, and risk-stratified model for SARS-CoV-2 transmission. The model, calibrated to the number of daily new cases reported across the 30 regions, relied on only five free parameters. It successfully captured the dynamics of SARS-CoV-2 trends (Supplementary Fig. S1), accurately reflecting three national peaks in infection levels and the subsequent variations in daily case rates. Additionally, the model illuminated periods of decline in daily case numbers and the duration of each stabilization phase (Fig. 1a). It also produced age and regional distributions of SARS-CoV-2 infections that aligned with the observed data (Fig. 1b,c). To account for underreporting due to asymptomatic cases or individuals with mild symptoms who did not seek medical care, we calibrated our model using three different age-specific estimates for the proportion of underreporting. These estimates range from 1 to 3 unreported cases for each reported case, corresponding to ratios of 1:3 (Fig. 1), 1:2 (Supplementary Fig. S3), and 1:1 (Supplementary Fig. S4).

### The value of information to improve vaccination strategies

**Figure 2:**
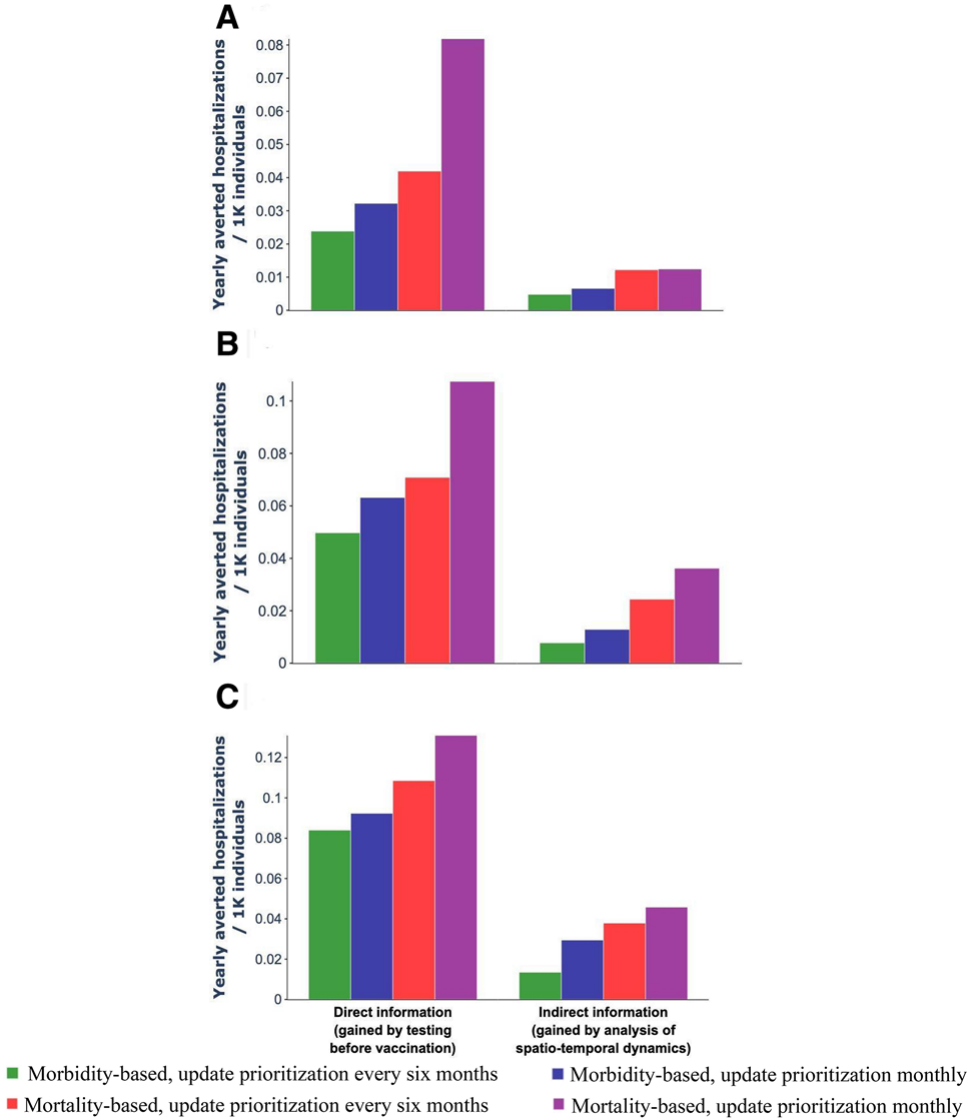
The effectiveness of vaccination strategies in reducing the yearly number of hospitalizations per 1000 individuals for different vaccine inventory levels and an estimated ratio of three unreported cases per reported case. Yearly number of averted hospitalizations (**a**–**c**) per 1000 individuals for vaccine inventory levels equivalent to 10%, (**a**) 15% (**b**), and 20% (**c**) of the entire population, over a time horizon of 3 years, relative to the baseline strategy, for each of the suggested strategies.

We simulated eight distinct booster vaccination administration strategies, varying in how they utilize direct and indirect information, as well as inventory levels (Fig. 2). Specifically, we explored all combinations of (1) vaccination orientation (morbidity-based vs. mortality-based strategy), (2) time elapsed since the last vaccination (using vs. not using information on vaccination timing), and (3) serological tests (using vs. not using direct information from pre-vaccination serological tests in vaccination decisions). Using projections from our calibrated model, we estimated the yearly number of hospitalizations that could be averted per 1000 individuals (Fig. 2). We set as the baseline, the strategy that targets population at high-risk for COVID-19 mortality (mortality-based strategy).

We found that strategies utilizing only indirect information could improve efficiency without additional costs, particularly in reducing hospitalizations. For instance, under assumptions of three unreported cases for every reported case, a low vaccine inventory level (10% of the total population), and the absence of serological tests, a morbidity-based strategy that leverages indirect information to determine vaccination timing could reduce hospitalizations per 1000 individuals by a factor of 0.005 (Fig. 2a). Furthermore, each tested strategy surpassed the baseline in terms of averted hospitalizations, underscoring the value of employing readily available indirect information, which incurs no additional costs.

The use of direct information reduces the number hospitalizations compared to strategies that only utilize indirect information. For instance, in a scenario with three unreported cases for every reported case, a medium vaccine inventory level (15% of the population), and accessible serological tests, a mortality-based strategy incorporating serological testing data can reduce hospitalizations per 1000 individuals by a factor of 0.11 (Fig. 2b).

Our results strongly support the integration of both indirect and direct information in vaccine administration strategies, as these data substantially lower the rates of hospitalizations and reported cases. This trend holds true across various vaccine inventory levels and different ratios of unreported to reported cases. To validate our findings, we further analyzed our proposed strategies' effectiveness in reducing annual reported cases per 1000 individuals (supplementary Figs. S5, S6, S7). Overall, among all tested scenarios, the morbidity-based strategy using both direct and indirect information showed the greatest reduction in the annual number of reported cases, while the mortality-based strategy using both types of information was most effective in reducing hospitalizations.

**Figure 3:**
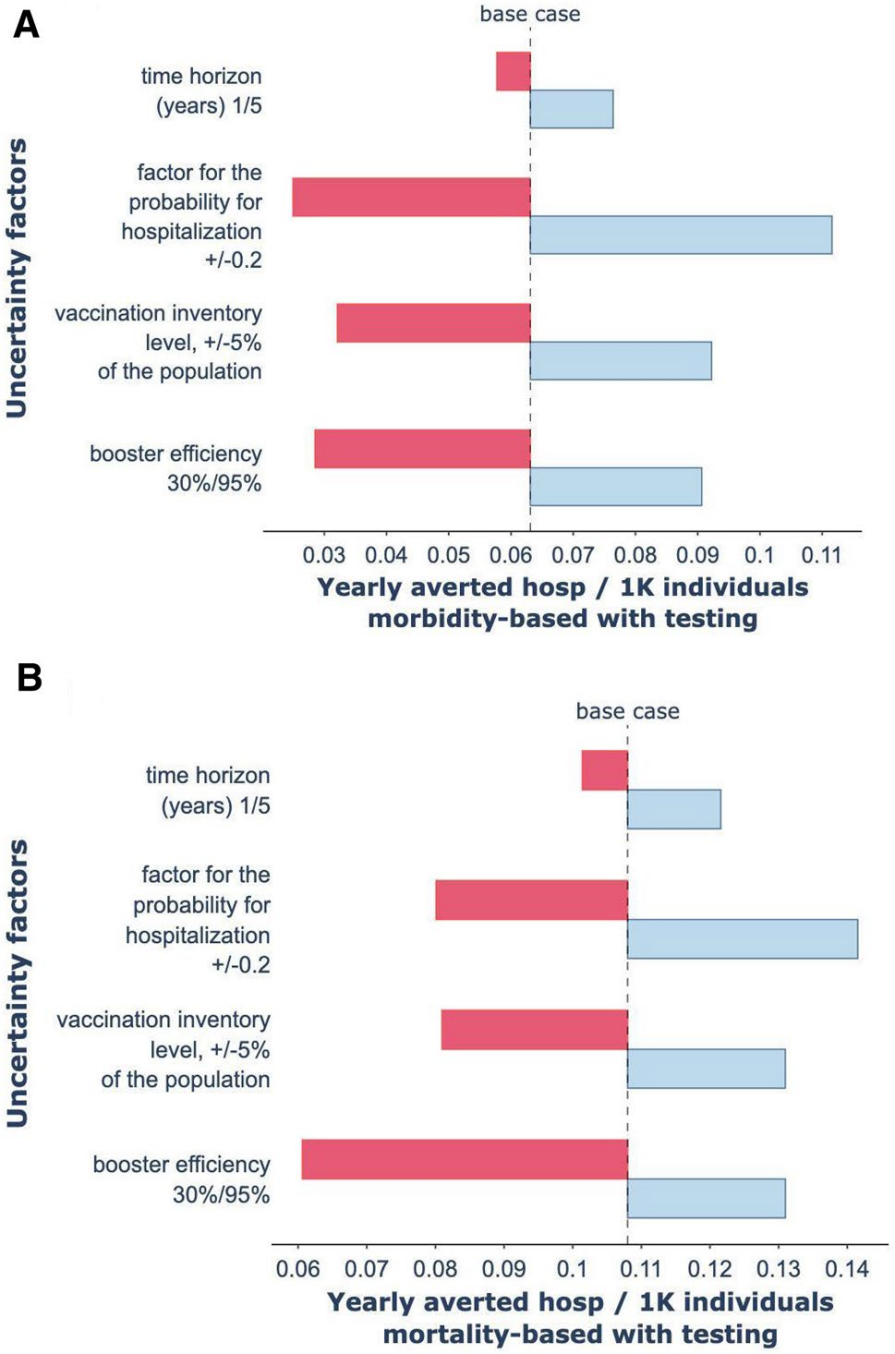
Univariate sensitivity analysis of the yearly averted hospitalizations per 1000 individuals. Yearly averted hospitalizations per 1000 individuals using morbidity-based approach (**a**) and mortality-based approach (**b**), relative to the baseline strategy. The considered underreporting rate is 1:3.

To ascertain the robustness of our findings, we conducted a univariate sensitivity analysis on the most effective strategies for reducing hospitalizations. This involved evaluating the number of yearly hospitalizarions averted per 1000 individuals for each selected strategy. Our analysis revealed that although variations in the factors of uncertainty quantitatively influence the outcomes, the overall conclusions remain consistent. For instance, under a mortality-based strategy utilizing both direct and indirect information, with a booster efficiency of 30%, we observed 0.061 averted hospitalizations per 1000 individuals annually (Fig. 3b). To further complement our analysis, we included results for the number of reported cases per 1000 individuals (Supplementary Fig. S8). These findings held true across different ratios of unreported versus reported cases (supplementary Figs. S9, S10).

**Figure 4:**
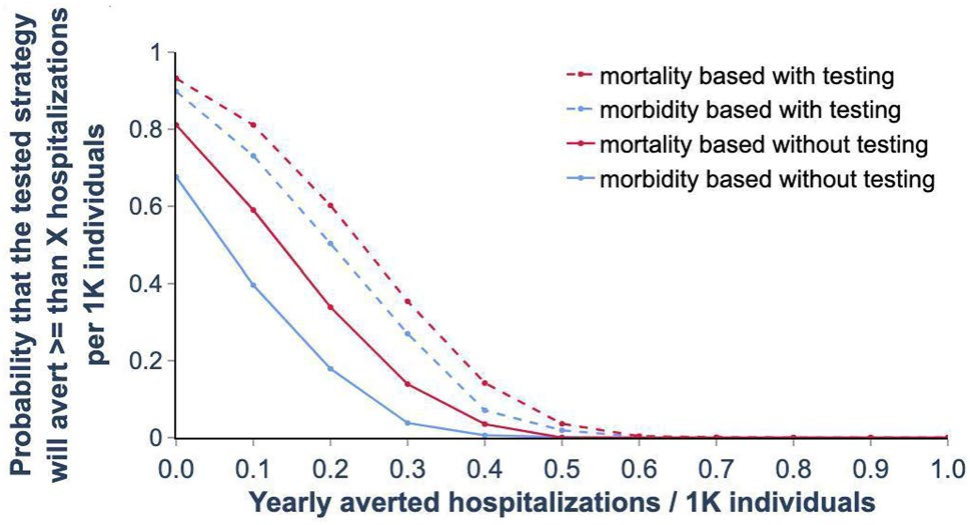
Global uncertainty analysis results. The threshold curves show the effect of a suggested strategy on the probability to avert an equal amount of hospitalizations per 1000 individuals per year, or higher, relative to the baseline strategy. The considered underreporting rate is 1:3.

We also performed a comprehensive global probabilistic uncertainty analysis. Our findings indicate that, even when considering all uncertainty factors collectively, mortality-based strategy that utilizes direct information is more effective in reducing hospitalizations than the rest of the considered strategies (Fig. 4). A similar analysis was conducted for the number of reported cases (Supplementary Fig. S11), and the results were consistent across various ratios of unreported to reported cases (Supplementary Figs. S12, S13).

## Discussion

Our key findings suggest that vaccination strategies based on direct insights into individual immunity levels from active serological testing, as well as indirect information obtained through sample-based sero-surveillance, or essential demographic, geographic, and epidemiological factors, can significantly reduce COVID-19 hospitalizations. The greatest reduction is achieved by incorporating serological tests into the vaccination strategy. This approach selectively targets individuals who lack adequate protection, thus optimizing vaccine distribution. By vaccinating only those who are mostly susceptible among the population at risk, this method allows policymakers to achieve a more efficient and targeted reach. The model, grounded in comprehensive data from Israel, demonstrates the effectiveness of this nuanced strategy in managing public health responses.

Should serological tests be out of reach, higher efficiency can be obtained even by only utilizing indirect information. This is achieved by offering booster vaccinations after shorter intervals (relative to the current policy) while considering the location of each segment of the population when prioritizing vaccination administration. We can indirectly conclude that following a surge in infection in a certain area, the population in the said area would be naturally immune to the virus. Under such circumstances, it would be more effective to prioritize locations with a low morbidity rate as a means to prevent the spread of COVID-19. Subsequently, time can be indirectly used to prioritize segments of the population who have not been recently vaccinated. This inference is based on the waning of antibodies, which causes a decrease in protection from natural immunity or vaccination.

Our findings are consistent under different inventory levels and unreported versus reported case ratios. To further confirm the robustness of our findings, we conducted sensitivity analyses that accounted for different time horizons, levels of vaccine effectiveness, probability of hospitalization, and vaccination inventory level. While changes in each parameter did alter the results quantitatively, all the conclusions remained stable. Even when considering all the uncertainty factors together, our suggested policies are preferable.

Our results show that serological testing is a prominent key in averting reported cases and hospitalizations. Although relatively expensive compared to other test methods, this cost may be countered by that savings in vaccination doses and related economic outcomes, such as loss in productivity. However, future research is essential to more precisely quantify the cost-effectiveness of serological testing. These studies should focus on meticulously evaluating the sensitivity and specificity of serological tests in determining immunity against prevalent COVID-19 variants. This approach will enable a more nuanced understanding of the tests' efficacy and economic viability in public health strategies.

As in any modelling study, we made several assumptions. Vaccination programs initiated by policymakers worldwide appeal to the whole population currently eligible for a vaccine dose by emphasizing the importance of preventing the virus from being spred^21^. Our model operates under the assumption that individuals will opt to be vaccinated if a booster shot is recommended. In practice, not all individuals adhere to vaccination recommendations, a factor that we accounted for in our assumption that the inventory level should not exceed more than 20% of the entire population.

We utilized a workplace parameter, taken from an available data source^22^, that describes the change in the number of visitors in workplaces since the beginning of the pandemic in Israel. Although correlated with the reported cases in Israel, this index is exposed to biases as it is influenced by holidays and local events and differs from one country to another. It is necessary to ensure this index contributes to the understanding of the spatio–temporal dynamics of the virus should it be implemented in different countries.

Social contact patterns are a crucial factor in the transmission of COVID-19 and other infectious agents in a population^23,24^. We did not consider changes in the contact patterns due to reported illness, including self-isolation. Thus, the effect of self-isolation was minimal and was not explicitly modelled. Additionally, changes in contact patterns due to lockdown and social distancing were not considered. Future studies could evaluate the effect of these measures on prioritizing segments of the population for vaccination administration to effectively avert reported cases and hospitalizations.

Our work demonstrates that to understand the spatio–temporal dynamics of transmission, models must account for risk- and age- groups as well as locations associated with sociodemographic factors. It also underscores the importance of considering behavioural aspects to accurately reflect the spatio–temporal dynamics of transmission. Although consisting of many compartments, our model is flexible and easy to understand, allowing modifications with minor changes. Our model is further applicable to many countries and scenarios, making it a useful, practical tool. However, there are several limitations to the use of the transmission model. Our behavioural parameters are limited in their ability to capture protective behaviour such as wearing a mask and maintaining physical distancing, as well as a population’s adherence to rules and instructions, which varied dramatically among the various lockdown periods. These observations are crucial to exploring transmission dynamics as they may vary considerably across subpopulations and change drastically during the pandemic. Thus, we note that our study identifies and relies on correlations and associations and does not attempt to assume causality.

Our transmission model relies on exponential distributions and, hence, is characterized by the memoryless property. As our model is unable to track recovered individuals, we cannot distinguish between recovered individuals after a symptomatic infection and those that experienced an asymptomatic infection. In practice, recovered individuals after a symptomatic infection will not acknowledge a vaccination administration. This observation underscores the potential of obtaining a higher reduction in reported cases and hospitalizations should our model be able to differentiate between these two subgroups.

Our approach may be generalized to optimize booster vaccination strategies for other infectious diseases, including influenza. Specifically, due to cross-reactive antibodies elicited by previous exposure^25–29^ and the observation that around 20% of individual infected with influenza are asymptomatic^25^, individuals might be partially of fully protected from infection for a given season but being unaware of it. Therefore, influenza presents as a potential candidate for exploring strategies that consider the immunity to seasonal circulating strains to determine an effective vaccination policy.

## Conclusion

We demonstrated that it is feasible to utilize aggregated, anonymized, and publicly available data in conjunction with direct information from active serological testing and indirect information inferred from the transmission dynamics of the virus to develop an adaptive and efficient booster vaccination strategy. Our transmission model predicts that, instead of adhering to a fixed mortality-based strategy for booster vaccination administration, employing both direct and indirect information can significantly reduce COVID-19 cases and hospitalizations while using the same number of vaccine doses. Even if serological tests are inaccessible or not cost-effective at accepted level, incorporating indirect information into the strategy formulation process can still effectively lower the number of COVID-19 cases and hospitalizations, without incurring additional costs.

## Methods

### Health data

We used publicly available, daily data on the number of COVID-19 reported cases, tests, and vaccinations (1st, 2nd, and 3rd doses) from the Israeli Ministry of Health from 15 May 2020 to 25 October 2021^30–33^. Individuals were considered to have contracted COVID-19 if they tested positive in an RT-PCR test. The dataset stratifies the entire Israeli population into 1642 statistical regions, as defined by the Israeli Central Bureau of Statistics. We mapped the 1642 statistical regions to 30 larger regions in order to neutralize the noise in the lower-level data, which allowed us to more accurately capture the COVID-19 spatio–temporal dynamics in Israel. For privacy purposes, statistical regions were included in our dataset only if they had accumulated at least 15 cases, tests, and vaccinations.

We computed daily new cases, new tests, and new vaccinations (1st, 2nd, and 3rd doses) from this data. To account for the difference in the portion of each age group’s contribution to the daily new cases, we aggregated the new cases by date and age group and normalized the outcome for each date.

### Transmission model

We developed a dynamic model for age-, risk- and region-stratified SARS-CoV-2 infection progression and transmission in Israel. Our model is a modified, advanced Susceptible-Exposed-Infected-Recovered (SEIR) compartmental framework^34^, where the population is stratified into health-related compartments and transitions between compartments change over time (Supplementary Fig. S1). To model age-dependent transmission, we stratified the population into age groups: 0–4 years, 5–9 years, 10–19 years, 20–29 years, 30–39 years, 40–49 years, 50–59 years, 60–69 years and ≥ 70 years. We distinguished between high-risk and low-risk individuals in each age group based on the ACIP case definition^35,36^. We also distinguished between the above-defined 30 regions covering Israel in the model.

Underreporting arises from asymptomatic cases or mild cases in individuals who do not seek care. Thus, following the early-infection phase, individuals in the model either transition to the infectious-and-reported compartment (${I}^{reported}$) or to the infectious-and-unreported compartment ${(I}^{unreported}$)^37,38^.

Multiple infections with SARS-Cov-2 occur due to waning immunity^39^, similarly to other respiratory infections^24,40^ Thus, we distinguish between two types of Susceptible-Exposed-Infected-Recovered (SEIR) compartmental frameworks: (1) individuals who have never been infected with SARS-CoV-2, (2) re-infected individuals due to immunity waning following infection or vaccination. Consistent with previous studies^11,40,41^, we assume that upon recovery individuals are fully, albeit temporarily, protected with a mean waning time of 9 months. Studies suggest that like with other respiratory infections, it is likely that if reinfection occurs, it is less severe and less transmissive^24,41^.

Evidence of antibody waning has motivated policymakers worldwide to initiate booster-shot programs to further enhance protection against SARS-CoV-2. Hence, we integrated three compartments to model the proportion of the population who received each of the vaccine doses, as follows: first dose ${V}_{j,k,r}^{1},$ second dose ${V}_{j,k,r}^{2}$ and booster dose ${V}_{j,k,r}^{booster}$.

Altogether, our model includes $13*9*2*30= 7020$ compartments ($health-compartments*age-groups*risk-groups*regions$).

### Force of infection

The rate at which individuals transmit SARS-Cov-2 at time t is ${\lambda }_{j,k}(t)$. This rate depends on the combination of (1) contact mixing patterns between an infected individual and his or her contacts, (2) age-specific susceptibility to infection, (3) interventions applied by the government, and (4) the presence of VOCs (variants of concern). We incorporate age- and region-specific contact patterns between individuals, represented by the contact rate between an infected individual in age-group $i$, region-group $l$ and each of their contacts who are susceptible in age-group $j$, region-group $k$, reflecting the contact rate in the pre-COVID-19 period. The contact matrix ${C}_{\left(l,i\right),(k,j)}$ is permanent (See Sect. 1.3 and the Supplementary Information Section Force of Infection).

The high regional variations in susceptibility were parameterized based on fertility rates and socioeconomic characteristics. The fertility rate in Israel correlates with the population density, household size, and SES^15^. The age-specific susceptibility rate of individuals, ${\beta }_{j}$, was parameterized by calibrating our model with daily COVID-19 incidents. Between 15 May 2020 and 25 October 2021, intermittent school closures were enforced to restrain the virus’s spread. This intervention was parameterized as ${\widehat{\beta }}_{school}$. During the aforementioned period, businesses were closed intermittently and isolation measures was enacted. To reflect the influence of these interventions as well as the populations’ adherence to them, we utilized a workplace parameter (${\widehat{\beta }}_{workplace}$), taken from an available data source^22^, describing the change in the number of visitors to workplaces since the beginning of the pandemic in Israel. The baseline value is defined as the median value for the 5-week period from 3 January 2020 to 6 February 2020. These parameters were calibrated to the epidemiological data of COVID-19 in Israel. To account for the prevalence of the B.1.1.7 and B.1.617.2 VOCs in Israel (Alpha variant and Delta variant, respectively), we explicitly considered in our model two parameters, reflecting the higher infectious rate relative to the wild-type SARS-Cov-2. These parameters influence the force of infection only during times when the VOCs were present in Israel. We utilized an additional parameter to account for the B.1.1.529 VOC (Omicron variant), to validate the projection of our calibrated model. Similarly, this parameter influences the force of infection only during the period when the B.1.1.529 variant was present in Israel.

### Model calibration

To empirically estimate unknown epidemiological parameters (Table S5), we calibrated our model to daily age-stratified cases of COVID-19 confirmed by PCR tests in 30 regions covering Israel. The calibration was conducted on the 30-region level to ensure there were sufficient time-series data points in each location for each age group. The data were reported by the Israeli Ministry of Health between May 2020 and October 2021 and contained daily information on the patients’ age, residential zone, underlying conditions, and clinical outcomes, including hospitalizations and death. To calibrate the model, we minimized the mean squared error (which is also the maximum likelihood estimation assuming the error is normally distributed) between the model projections of reported cases and the daily COVID-19-confirmed cases’ data. Due to the uncertainty regarding the proportion of unreported cases, we calibrated our model to different scenarios. Specifically, underreporting is affected by the testing policy and testing capabilities of each country, as well as individuals' tendency to seek care once clinical symptoms appear and severity of the infection, which is associated with age^42^. We, therefore, chose different age-specific estimates for the proportion of underreporting, ranging from 1 to 3 unreported cases per reported case. These estimates are based on a meta-analysis of 77 unique studies conducted worldwide to determine the percentage of asymptomatic infections among the confirmed population. To account for the age variation, we considered the detailed results of this meta-analysis for each age group^43^.

The final transmission model included five parameters without constraints imposed from previous data: reduced susceptibility due to school closure ${\widehat{\beta }}_{school},$ reduced contact mixing patterns due to isolation provision and businesses closure ${\widehat{\beta }}_{workplace}$ and susceptibility rate based on age groups *j*: 0–19, 20–59, and > 60.

We validated our calibrated model by using its projections from 25 October 2021, until the end of the presence of the B.1.1.529 VOC (Omicron variant), 18 March 2022.

### Model simulations

We evaluated the effectiveness of utilizing serological tests, location, and time since the last vaccination administration in forming a vaccination strategy that reduces the number of reported cases and hospitalizations. The current strategy used by the policymakers in Israel serves as the “baseline” strategy. This strategy is a simple, fixed mortality-based strategy that considers only age- and risk- groups and does not utilize direct or indirect information. We proposed eight different booster vaccination strategies, considering intermittently all combinations of (1) vaccination orientation (morbidity-based vs. mortality-based strategy), (2) time from last vaccination (utilizing vs. not utilizing information on the timing of vaccination administration), and (3) serological tests (utilizing vs. not utilizing direct information from serological tests conducted prior to vaccination in the decision to vaccinate individuals). All the explored strategies consider geographic location in their decision-making process. In each simulation, and before every vaccination administration, we created a priority queue to determine which segments of the population should be vaccinated first. We vaccinated according to this prioritization, up to the point where all the supply of allocated vaccinations is administered.

According to the current strategy’s vaccination administration timing, the population is offered a booster vaccine dose every 6 months. To utilize the indirect information derived from time, we checked a uniform vaccination methodology where 1/6 of the original vaccination inventory (which is replenished every 6 months) administered every month. Using this methodology, we take advantage of the transmission model and prioritize the vaccinations in an adaptive manner.

Recent studies suggest that individuals who were previously infected with SARS-CoV-2 gain additional protection from a subsequent single-dose vaccine regimen (namely, a booster dose)^41,44^. Hence, a booster vaccine is offered to all individuals in Israel whose PCR tests did not come back positive. This indiscriminate vaccination approach leads to the vaccination of recovered yet unreported patients due to asymptomatic reactions or lack of interest in seeking care, i.e., individuals with sufficient antibody protection against the virus. To evaluate the ineffectiveness of this approach, we compared two strategies: (1) vaccination strategy based on information about the recovered yet unreported cases and (2) vaccination strategy that did not incorporate information about the recovered yet unreported cases. Information on recovered yet unreported cases can be obtained directly from serological testing, which provides and immediate indication of the level of antibodies present in the circulation. When this information is available, we do not take into account the recovered-yet-unreported proportion of the population when calculating the number of administered vaccines. When this information is not available, we include the recovered-yet-unreported proportion of the population when calculating the number of administered vaccines.

Our evaluation of these strategies in terms of their ability to reduce the number of reported cases and hospitalizations was achieved by simulating the model for 3 years. For each strategy, we examined 3 different booster vaccination inventory levels: equal to (1) 10% (low), (2) 15% (medium), and (3) 20% (high) of the entire population. This evaluation was conducted separately for reported cases and for hospitalizations. The inventory was renewed every 6 months. To ensure the robustness of our results, the evaluation was performed for three different underreporting ratios: 1:1, 1:2, and 1:3 unreported versus reported cases. For each scenario, we computed the annual number of averted reported cases and hospitalizations per 1000 individuals relative to the baseline strategy, using the model’s projections. For example, the number of averted hospitalizations per 1000 individuals per year was calculated by subtraction the annual number of hospitalizations per 1000 individuals for each of the two strategies from the annual number of hospitalizations per 1000 individuals for the baseline strategy (Fig. 2).

### Sensitivity analyses

The following four uncertainty factors were selected: (1) time horizon (1–5 years), (2) booster dose effectiveness (30–95%), (3) probability of hospitalization (0.8–1.2) and (4) vaccination inventory level (10–20% of the entire population). The robustness of the results was evaluated on the predominant strategy to avert reported cases and the predominant strategy to avert hospitalizations. A univariate sensitivity analysis was performed for each combination of strategy and health measurement using the four uncertainty factors. The number of averted cases or hospitalizations was defined relative to the baseline strategy received in the simulation’s result as the base case. The absolute difference value was computed from the base case to the number of averted cases or hospitalizations obtained from simulating both the baseline strategy and the proposed strategy while changing only one uncertainty factor intermittently for both ends (Fig. 3, and supplementary Figs. S8–S10).

A global probabilistic sensitivity analysis was performed for both health measurements while considering only 4 proposed strategies utilizing the indirect information inferred from the time since last vaccination administration. In this analysis, values were randomly selected from the domain of each of the uncertainty factors and incorporated into the settings of the baseline strategy alongside the proposed strategies, to compute the yearly number of total averted reported cases or hospitalizations per 1000 individuals. This calculation was then used to examine the probability that the tested strategies avert a certain amount (or higher) of yearly reported cases or hospitalizations per 1000 individuals (Fig. 4 and supplementary Figs. S11–S13).

### Supplementary Information

Supplementary Information.

## Data Availability

All data used in this paper are publicly available. Statistical code will be available after acceptance.
