# Supplementary material for: Utilizing direct and indirect information to improve the COVID-19 vaccination booster scheduling
Source: Sci Rep. 2024 Apr 6;14:8089. doi: 10.1038/s41598-024-58690-8 (PMC10998875; doi:10.1038/s41598-024-58690-8)
Supplement: Supplementary file 1 — Supplementary Information. [file 41598_2024_58690_MOESM1_ESM.docx]

**Supplementary information for: Utilizing direct and indirect information to improve the COVID-19 vaccination booster scheduling**

Yotam Dery^1,¶^, Matan Yechezkel^1^, Irad Ben-Gal^2^, Dan Yamin^1,3,*^

^1^ Laboratory for Epidemic Modeling and Analysis, Department of Industrial Engineering, Faculty of Engineering, Tel Aviv University, Tel Aviv 6997801, Israel

^2^ Laboratory for AI, Machine Learning & Business Data Analytics, Department of Industrial Engineering, Faculty of Engineering, Tel Aviv University, Tel Aviv 6997801, Israel

^3^ Center for Combatting Pandemics, Tel Aviv University, Tel Aviv 6997801, Israel

* To whom correspondence should be sent: Dan Yamin, PhD Email: [dan.yamin@gmail.com](mailto:dan.yamin@gmail.com)

**Table of Content**

[1. **Model** 2](#_Toc156228041)

[1.1. **The model** 2](#_Toc156228042)

[1.2. **Model transitioning** 6](#_Toc156228043)

[1.3 **Force of infection** 8](#_Toc156228044)

[2. **Fixed parameters** 9](#_Toc156228045)

[2.1. ***Contact matrix*** 9](#_Toc156228046)

[2.2. ***1^st^ and 3^rd^ vaccination administration Contact matrix*** 10](#_Toc156228047)

[2.3. **Epidemiological parameters** 11](#_Toc156228048)

[3. **Calibrated parameters** 16](#_Toc156228049)

[4. **Further results** 19](#_Toc156228050)

[4.1. **Model calibration** 19](#_Toc156228051)

[4.2. **Model simulations** 22](#_Toc156228052)

29

[5. **References** 33](#_Toc156228054)

1. **Model**
   1. **The model**

We developed a dynamic model for age-, risk- and regions-stratified SARS-Cov-2 infection progression and transmission in Israel. Our model is a modified Susceptible-Exposed-Infected-Recovered (SEIR) compartmental framework^1^, whereby the population is stratified into health-related compartments, and transitions between the compartments occur over time (Fig. S1). To model age-dependent transmission, we stratified the population into nine age groups: 0–4 years, 5-9 years, 10-19 years, 20-29 years, 30-39 years, 40-49 years, 50-59 years, 60-69 years and ≥70 years. ^2–4^. We distinguished between high-risk and low-risk individuals for each age group based on the ACIP case definition^5,6^. We also distinguish in the model between 30 regions covering Israel in the model.

Vaccination is widely accepted as the most prominent measure in the fight against COVID-19, posing the greatest hope for ending this major global health pandemic and related economic crisis^7,8^. Consequently, an unprecedented international effort by private and public institutions alike was directed at accelerating the traditionally lengthy vaccine-development process^9–11^.
On 20 December 2020, Israel launched its COVID-19 vaccination campaign and administered more BNT162b2 vaccine doses than all countries aside from China, the US, and the UK. Moreover, Israel had administered almost 11.0 doses per 100 population, while the next highest rates were 3.5 (in Bahrain) and 1.4 (in the United Kingdom)^12^. This vaccination campaign has led Israel to have one of the highest rates of vaccinated individuals per capita, with 68.7%, 48% and 8% of the population having received the first or the second BNT162b2 vaccine or having recovered from Covid-19, respectively, as of 24 February 2020, taking into account population older than 16 years - the population currently eligible for vaccination^13^. On 30 July 2021, In light of the surge in Covid-19 reported cases caused by the Delta (B.1.617.2) variant of concern (VOC) in Israel^14–16^, a third (“booster”) dose of Pfizer-BioNTec’s BNT162b2 vaccine was administered for individuals 60 years or older^15,17^ and gradually expanded to 12 years or older^18^. Hence, our model allocates three compartments to represent the three doses of COVID-19 vaccine that were given in Israel as follows: first dose $V_{j,k,r}^{1}\left( t \right),$second dose $V_{j,k,r}^{2}\left( t \right)$ and third dose $V_{j,k,r}^{3}(t)$.

Multiple infections with SARS-Cov-2 occur due to the waning of antibodies^19^, similarly to other respiratory infections^20,21^ Studies found that similarly to other respiratory infections, it is likely that if re-infection occurs, it is less severe and less transmissive^20,21^. A recent study suggests that Individuals who were previously infected with SARS-CoV-2 seem to gain additional protection from a subsequent single-dose vaccine regimen, such that vaccinated individuals are at a 5.96-fold increased risk for breakthrough infection and at a 7.13-fold increased risk for symptomatic disease, compared to those previously infected^16,22^. vaccinated individuals were also at greater risk for COVID-19-related-hospitalization compared to those who were previously infected^16^. These findings correspond to previous reports^21,23,24^.

Thus, we distinguish between two types of Susceptible-Exposed-Infected-Recovered (SEIR) compartmental frameworks: while the first framework designates individuals who have never been infected with SARS-CoV-2, the second illustrates re-infections that may occur due to waining period after recovery or decrease in vaccine efficiency. Consistent with previous studies^16,21,25^ we assumed that upon recovery individuals are fully, albeit temporarily, protected with a mean waning of 9 months.

Underreporting arises from asymptomatic cases or mild cases of individuals that do not seek care^26–29^. Thus, following the early exposure phase, individuals in the model transition either to an infectious and reported compartment $I^{reported}$, or to infectious and unreported compartment $I^{unreported}$, on both of the compartmental frameworks.

Accordingly, we stratified the population into thirteen health-related compartments: susceptible$S_{j,k,r}\left( t \right)$, exposed but not yet infectious$E_{j,k,r}\left( t \right)$, reported infectious$I_{j,k,r}^{reported}\left( t \right)$, unreported infectious $I_{j,k,r}^{unreported}\left( t \right)$ and recovered $R_{j,k,r}\left( t \right)$. These group of compartments repeats itself twice (denoted with 1 for the first group and 2 for the second group), to support multiple infections with SARS-Cov-2, such that in any given time t (in days) the population is fixed and scaled to one. Namely,

|  | $\sum_{j} \sum_{k} \sum_{r} \left[ S_{j,k,r}^{1}\left( t \right)+ E_{j,k,r}^{1}\left( t \right)+ I_{j,k,r}^{reported,1}\left( t \right)+I_{j,k,r}^{unreported,1}\left( t \right)+R_{j,k,r}^{1}\left( t \right)+S_{j,k,r}^{2}\left( t \right)+ E_{j,k,r}^{2}\left( t \right)+ I_{j,k,r}^{reported,2}\left( t \right)+I_{j,k,r}^{unreported,2}\left( t \right)+R_{j,k,r}^{2}\left( t \right)+V_{j,k,r}^{1}\left( t \right)+V_{j,k,r}^{2}\left( t \right)+V_{j,k,r}^{3}\left( t \right) \right]= \sum_{j} \sum_{k} \sum_{r} N_{j,k,r} =1,$ | (1) |
| --- | --- | --- |

where the index $j\in\{0-4y,5-10y,\ldots,>70y\}$ represents the age-group of each individual, index $k\in\left\{ 1,2,\ldots,30 \right\}$ specifies the home region of each individual and index $r\in\{L,H\}$ specifies the risk-group of each individual (i.e. High-risk, or low-risk). Altogether, our model includes $13*9*2*30= 7,020$


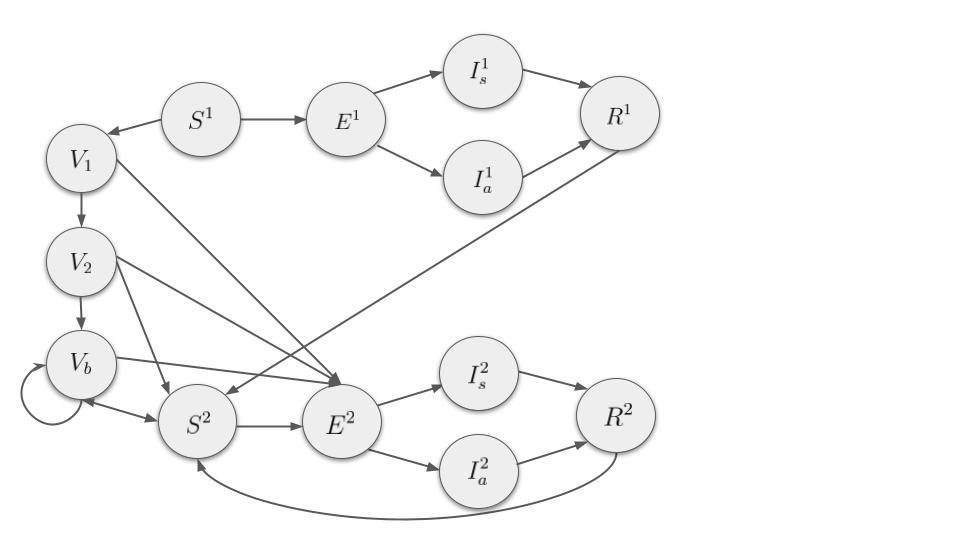


**Fig. S1**. **A compartmental diagram to describe the structure of the transmission model.** Susceptible individuals in $S^{1}$ transition to the exposed compartment with a force of infection λ, where they are infected as a result from contact with an infectious individual. Infected individuals move to a reported *I^Reported,1^* or unreported *I^Unreported,1^* infectious period, in which they may have a mild or an asymptomatic infection until death or complete recovery. Due to waning of antibodies, individuals return to a susceptible phase $S^{2}$ from which they may administer for a booster dose or go through a similar process as described above, for unlimited number of subsequent infections. Susceptible individuals in $S^{1}$ may transit to $V_{1}$ should they administered with 1^st^ vaccine dose, where they move to $V_{2}$ in a mean time of 21 days. Should they be found eligible for a booster dose, they can move to $V_{b}$ upon administration of the vaccine. They might stay in this compartment in case of subsequent booster vaccination administration, transit to $S_{2}$ due to waning of antibodies or move to $E_{2}$ in case of contact with infectious individual and failure in protection granted by the vaccine, like early vaccinated compartments. For clarity of depiction, age, risk, and region stratifications are not displayed.

- 1. **Model transitioning**

Susceptible individuals which had never been infected $S_{j,k,r}^{1}\left( 0 \right)$, transition to two different compartments: exposed compartment $E_{j,k,r}^{1}(t)$, with the force of infection $\lambda_{j,k}(t)$, depending on their age-group *j* home region-group *k,* or vaccinated-with-first-dose compartment $V_{j,k,r}^{1}\left( t \right)$ at rate $\eta_{j,r}$, depending on their age-group *j* and risk-group *r.*

Considering an encounter of susceptible and infected individuals, infection occurs at rate $\delta$, leading to either an unreported case (having non to mild symptoms) with probability $f_{j,r}$, which results in a transition to $I_{j,k,r}^{unreported,1}(t)$, or a reported case (having moderate to severe symptoms) with probability of $(1-f_{j,r})$ which results in a transition to $I_{j,k,r}^{reported,1}(t)$.

After the infectious period, individuals transition into the recovered compartment at rate $\gamma$, $R_{j,k,r}^{1}(t)$. Due to the waning of antibodies, individuals transition to $S_{j,k,r}^{2}(t)$ at a mean time of nine months, from which they go through a similar process as described above, for an unlimited number of subsequent infections. The vaccination protocol in Israel corresponds to Pfizer BioNTech guidelines. Consequentially, the recommended interval between the first and the second doses is 21 days^30^. Hence, first-dose-vaccinated individuals transition to $V_{j,k,r}^{2}\left( t \right)$at a rate of $\frac{1}{21}$­_days­_, and move to ${V_{b}}_{j,k,r}\left( t \right)$ with a rate of $\kappa_{j,r}$, depending on their age-group *j* home region-group *k.* As individuals are not fully protected against the virus upon each vaccination administration, they may transit to the exposed compartment $E_{j,k,r}^{2}(t)$ at a rate of $\lambda_{j,k}\left( t \right)*(1-V_{effectiveness})$, depending on their age-group *j,* home region-group *k* and the effectiveness of the recent administered dose. Recovered individuals currently in $S_{j,k,r}^{2}\left( t \right)$ can transit to ${V_{b}}_{j,k,r}\left( t \right)$

At a rate of $\kappa_{j,r}$ upon booster dose administration. (See Section, 2.3 Epidemiological parameters).

Thus, the transmission model is composed of the following system of difference equations:

|  | $\Delta S_{j,k,r}^{1}\left( t \right)=-\lambda_{j,k}{*S^{1}}_{j,k,r}\left( t-1 \right)-\eta_{j,k}{*S^{1}}_{j,k,r}\left( t-1 \right)$ ${\Delta E}_{j,k,r}^{1}\left( t \right)=\lambda_{j,k}{*S^{1}}_{j,k,r}\left( t-1 \right)-\delta{*E}_{j,k,r}^{1}\left( t-1 \right)$  ${\Delta I_{s}^{1}}_{j,k,r}\left( t \right)=\left( 1-f_{j} \right)*\delta*E_{j,k,r}^{1}\left( t-1 \right)-\gamma_{j,r}*{I_{s}^{1}}_{j,k,r}\left( t-1 \right)$  ${{\Delta I}_{a}^{1}}_{j,k,r}\left( t \right)=f_{j}*\delta*E_{j,k,r}^{1}\left( t-1 \right)-\gamma_{j,r}{{*I}_{a}^{1}}_{j,k,r}\left( t-1 \right)$  ${\Delta R}_{j,k,r}^{1}\left( t \right)=\gamma_{j,r}*\left( {I_{s}^{1}}_{j,k,r}\left( t-1 \right)+{I_{a}^{1}}_{j,k,r}\left( t-1 \right) \right)-\omega*R_{j,k,r}^{1}(t-1)$  $\Delta{V_{1}}_{j,r,k}\left( t \right)= \eta_{j,k}*S_{j,k,r}^{1}\left( t-1 \right)-\zeta\boldsymbol{*}V_{1_{j,k,r}}(t-1) -\lambda_{j,k}*\left( 1-{V_{1}}_{eff} \right){*V}_{1_{j,k,r}}(t-1)$  $\Delta{V_{2}}_{j,k, r}\left( t \right)= \zeta\boldsymbol{*}V_{1_{j,k,r}}\left( t-1 \right)-\lambda_{j,k}*\left( 1-{V_{2}}_{eff} \right)*V_{2_{j,k,r}}\left( t-1 \right)-\tau*V_{2_{j,k,r}}\left( t-1 \right)-\kappa_{j,k}*V_{2_{j,k,r}}\left( t-1 \right)$  $\Delta{V_{b}}_{j,k,r}\left( t \right)=$ $\kappa_{j,k}*{(V}_{2_{j,k,r}}\left( t-1 \right)+ S_{j,k,r}^{2}\left( t-1 \right))-\omega* {V_{b}}_{j,k,r}\left( t-1 \right)- \lambda_{j,k}*\left( 1-{V_{b}}_{eff} \right)$  $\Delta S_{j,k,r}^{2}\left( t \right)=$ $\omega*\left( R_{j,k,r}^{1}\left( t-1 \right)+R_{j,k,r}^{2}\left( t-1 \right) \right)+\tau*V_{2_{j,k,r}}\left( t-1 \right)+\omega* {V_{b}}_{j,k,r}\left( t-1 \right)-\kappa_{j,k}*S_{j,k,r}^{2}\left( t-1 \right)-\lambda_{j,k}*E_{j,k,r}^{2}\left( t-1 \right)$ ${\Delta E}_{j,k,r}^{2}\left( t \right)=\lambda_{j,k}*(S_{j,k,r}^{2}\left( t-1 \right)+\left( 1-{V_{1}}_{eff} \right){*V}_{1_{j,k,r}}\left( t-1 \right)+\left( 1-{V_{2}}_{eff} \right)*V_{2_{j,k,r}}\left( t-1 \right)+\left( 1-{V_{b}}_{eff} \right)*V_{3_{j,k,r}}\left( t-1 \right))-\delta{*E}_{j,k,r}^{2}\left( t-1 \right)$ ${\Delta I_{s}^{2}}_{j,k,r}\left( t \right)=\left( 1-f_{j} \right)*\delta{*E}_{j,k,r}^{2}\left( t-1 \right)-\gamma_{j,r}{{*I}_{s}^{2}}_{j,k,r}\left( t-1 \right)$  ${{\Delta I}_{a}^{2}}_{j,k,r}\left( t \right)=f_{j}*\delta{*E}_{j,k,r}^{2}\left( t-1 \right)-\gamma_{j,r}*{I_{a}^{2}}_{j,k,r}\left( t-1 \right)$  ${\Delta R}_{j,k,r}^{2}\left( t \right)=\gamma_{j,r}\left( {I_{s}^{2}}_{j,k,r}\left( t-1 \right)*{I_{a}^{2}}_{j,k,r}\left( t-1 \right) \right)-\omega*R_{j,k,r}^{2}(t-1)$  with initial conditions:  $S_{j,k,r}^{1}\left( 0 \right)= N_{j,k,r}$  $E_{j,k,r}^{1}\left( 0 \right)=I_{j,k,r}^{1, reported}\left( 0 \right)=I_{j,k,r}^{1,unreported}\left( 0 \right)=R_{j,k,r}^{1}\left( 0 \right)=0$*.* | (2) |
| --- | --- | --- |

1.3 **Force of infection**

The rate at which individuals transmit SARS-Cov-2 at time t is $\lambda_{j,k}(t)$. This rate depends on the combination of (i) contact mixing patterns between an infected individual and his or her contacts, (ii) age-specific susceptibility to infection, (iii) region-based behavioral susceptibility and (iv) presence of VOCs.

We incorporate age- and region-specific contact patterns between individuals, represented by the contact rate between an infected individual in age-group $i$, region-group $l$ and each of their contacts with susceptible in age-group $j$,region-group $k$. The contact matrix $C_{\left( l,i \right),(k,j)}$ is an average of three matrices, differ in their contact rate between an infected individual in age-group $i$, region-group $l$ and each of their contacts with susceptible in age-group $j$,region-group $k$, for different locations: at home, at work, and during leisure, as defined in a recent study^31^.

The high regional variations in susceptibility were parameterized based on fertility rates and socioeconomic characteristics relative to the national average, using the data from the Central Bureau of Statistics (CBS), $\alpha_{k}$. According to a previous study, regions with high fertility rates had less adhered to the mobility restrictions that were lifted in Israel, which was reflected by our model in higher susceptibility^31^. Between May 15, 2020, to October 25, 2021, intermittent school closures were carried out to restrain the virus. this intervention was parameterized as $\hat{\beta}_{school}$. During the aforementioned period businesses were closed intermittently, and provision of isolation was applied. To reflect the effect of these interventions as well as the populations’ adherence, we utilized a workplace parameter (denoted by $\hat{\beta}_{workplace}$) taken from an available data source^32^, describing the change of visitors in workplaces since the beginning of the pandemic in Israel. The baseline value is defined as the median value for the 5-week period from January 3 to February 6, 2020, in Israel. These parameters were calibrated to the epidemiological data of COVID-19 in Israel. To account for the prevalence of different VOCs appearance in Israel, B.1.1.7 and B.1.617.2 (Alpha variant and Delta variant respectively), we explicitly considered in our model two parameters, reflecting the higher infectious rate relative to the wild-type SARS-Cov-2. these parameters influence the force of infection only at times in which the VOCs were present in Israel. We utilized an additional parameter to account for the BA.1. VOC (Omicron variant) to validate the projection of our calibrated model. Similarly, this parameter influences the force of infection only at times in which the B.1.1.529 variant was present in Israel.

|  | $\lambda_{j,k}\left( t \right)= \left( {{a_{var}*d_{var}*o_{var}* (\hat{\beta}}_{activity}*activity\_rate)*\hat{\beta}_{school}*\hat{\beta}}_{j}*\theta_{k}*\left[ \sum_{i\in Age} \sum_{l\in Area} C_{\left( l,i \right),(k,j)}\sum_{\tau=1}^{2} \sum_{r\in Risk} \left( I_{i,l,r}^{reported,\tau}\left( t-1 \right)+I_{i,l,r}^{unreported,\tau}\left( t-1 \right) \right) \right] \right)$ | (3) |
| --- | --- | --- |

Taken together, the force of infection $\lambda_{j,k}(t)$ is given by:

1. **Fixed parameters**
   1. ***Contact matrix***

As transmission occurs upon a contact between individuals, our contact matrix constitutes an intrinsic component in our transmission model. this matrix describes the contact rate between an infected individual in age-group $i$, region-group $l$ and each of their contacts with susceptibility in age-group $j$,region-group $k$. The contact matrix $C_{\left( l,i \right),(k,j)}$ is an average of three matrices, differ in their contact rate between an infected individual in age-group $i$, region-group $l$ and each of their contacts with susceptible in age-group $j$,region-group $k$, for different locations: at home, at work and during leisure, as defined in a recent study^31^.

- 1. ***1^st^ and 3^rd^ vaccination administration Contact matrix***

We utilized publicly available, daily data on accumulated COVID-19 cases, tests, and vaccinations (1^st^, 2^nd^, and 3^rd^ doses) from the Israeli Ministry of Health from May 15, 2020, to October 25, 2021^33–36^. The dataset is stratified by date, 1,642 statistical regions comprising Israel as defined by the Israeli Central Bureau of Statistics, and by 10 age groups. For each date, we mapped the 1,642 statistical regions to 30 regions covering Israel, and the 10 age groups to nine age groups as defined in our model. For each date, we computed the rate of 1^st^ dose and 3^rd^ dose vaccination administration by dividing the number of vaccination administrations per region and age group by the number of eligible individuals per region and age group. These rates were incorporated in our transmission model as the transition rates to ${V_{1}}_{j,r,k}$ and ${V_{b}}_{j,r,k}$ respectively.

2.3. **Epidemiological parameters**

*Unreported cases*

Underreporting arises from asymptomatic cases or mild cases of individuals that do not seek care. The severity of SARS-Cov-2 infection is associated with age- and risk- group^37^. In addition, underreporting is affected by testing policy and testing capabilities for each country, as well as the tendency of individuals to seek care once clinical symptoms appear. As testing capabilities improve over time and tests become more scalable^38^, a large amount of data is accumulated regarding unreported cases. Thus, we evaluated unreported cases based on a meta-analysis that considers 77 different studies, conducted on 4 different continents and 77 different countries. The study suggests that each reported case corresponds to ~1 unreported case^39^. Other studies suggest that each reported case corresponds to ~5.5 unreported cases^29,40^, whereas estimates from the CDC suggest 4 unreported cases for a single reported case in the United States^41^. Taken together we chose to present estimates of unreported ratios 1:1 (Scenario A), 1:2 (Scenario B), and 1:3 (Scenario C).

We estimated the proportion of underreporting for each age-group by scaling the estimates from meta-analysis study to the age groups as defined in our model. This scaling suggested that younger age-groups are more likely to be unreported. Using these estimates we obtained that the overall proportion of unreported cases is 50% for scenario A, 67% for scenario B, and 75% for scenario C.

**Table S1.** **proportion of unreported cases.** The proportion of unreported cases among individuals stratified by age and overall reported cases based on previous studies.

| Scenario | Age | 0-19 | 20-39 | 40-59 | +60 |
| --- | --- | --- | --- | --- | --- |
| A |  | 0.602 | 0.494 | 0.324 | 0.338 |
|  | **Total** | 0.5 |  | | |
| B | **Low** | 0.714 | 0.637 | 0.515 | 0.525 |
|  | **Total** | 0.67 |  | | |
| C | **Low** | 0.816 | 0.766 | 0.687 | 0.694 |
|  | **Total** | 0.75 |  | | |

*Case hospitalization*

The probability of hospitalization for each age-and risk-group given a reported case was evaluated based on the Israeli Ministry of Health case report data (Table S2).

**Table S2**. **Probability of hospitalization for each age-and risk-group given a reported case**

| Age-group | Risk-group | Probability of hospitalization given a reported case |
| --- | --- | --- |
| 0-4 | **High**  **Low** | 0.029  0.006 |
| 5-9 | **High**  **Low** | 0.025  0.006 |
| 10-19 | **High**  **Low** | 0.025  0.006 |
| 20-29 | **High**  **Low** | 0.045  0.019 |
| 30-39 | **High**  **Low** | 0.114  0.027 |
| 40-49 | **High**  **Low** | 0.137  0.032 |
| 50-59 | **High**  **Low** | 0.163  0.038 |
| 60-69 | **High**  **Low** | 0.185  0.044 |
| +70 | **High**  **Low** | 0.208  0.050 |

*Variants of concern (VOCs)*

Over the course of time, new variants of SARS-Cov-2 emerged nationwide^42^, causing a surge in infections and severe diseases^43,44^. Variant B.1.1.7 (Namely, variant “Alpha” ) emerged in December 2020 in Israel, and endured until April 2021 ^45^, estimated to be more contagious than the wild type by 30-50%^46^. Variant B.1.617.2 (Namely, variant “Delta” ) prevailed from June 2021 until November 2021^47^ and was estimated to be 50% more transmissive than the Alpha variant. It was in December 2021 when Israel’s ministry of health tightened the isolation provision in light of several new reported cases caused by the B.1.1.529 variant (Namely, variant “Omicron”) which lasted until March 2022^48^. The BA.1 variant was estimated to be 2.4 times more transmissive than the Delta variant^49^.

We utilized these observations such that each variant increased the force of infection (see section 1.3) in the time frame it was present. We multiplied the force of infection by the transmissibility rate of the correspondent variant of concern to reflect the higher susceptibility of the population.

**Table S3**. **Fixed parameters used in the transmission model.**

| Parameter | Description | Value | Reference |
| --- | --- | --- | --- |
| $\boldsymbol{N}_{\boldsymbol{j}\boldsymbol{,}\boldsymbol{k}\boldsymbol{,}\boldsymbol{r}}$ | Population size of risk-group r age-group j in region k | Varies between regions | ^5^ |
| $\frac{\boldsymbol{1}}{\boldsymbol{\sigma}}$ | Mean duration of exposed period | $4.1 days$ | ^50–53^ |
| $\boldsymbol{f}_{\boldsymbol{j}\boldsymbol{,}\boldsymbol{r}}$ | Unreported probabilities | Table S1 | ^26,27,37^ |
| $\frac{\boldsymbol{1}}{\boldsymbol{\gamma}_{\boldsymbol{j}\boldsymbol{,}\boldsymbol{r}}}$ | Mean duration of late infectious period (in reported and unreported cases) | $7 days$ | ^52^ |
| $\frac{\boldsymbol{1}}{\boldsymbol{\zeta}}$ | Mean duration of time between 1^st^ dose and 2^nd^ dose | 21 days | ^30^ |
| $\frac{\boldsymbol{1}}{\boldsymbol{\omega}}$ | Mean duration of waning time | 270 days | ^16,25,54^ |
| $\boldsymbol{C}_{\left( \boldsymbol{l}\boldsymbol{,}\boldsymbol{i} \right)\boldsymbol{,(}\boldsymbol{j}\boldsymbol{,}\boldsymbol{k}\boldsymbol{)}}$ | Contact rate between an infected individual in age-group $i$, region-group $l$ and each of their contacts with susceptible in age-group $j$,region-group $k$. |  | ^55,56^‬‬‬‬‬‬‬‬‬‬‬‬‬‬‬‬‬‬‬‬‬‬‬‬‬‬‬‬‬‬‬‬‬‬‬‬‬‬‬‬‬‬‬‬‬‬‬‬‬‬‬‬‬‬‬‬‬‬‬‬‬‬‬‬‬‬‬‬‬‬‬‬‬‬‬‬‬‬‬‬‬‬‬‬‬‬‬‬‬‬‬‬‬‬‬‬‬‬‬‬‬‬‬‬‬‬‬‬ |
| $\boldsymbol{\alpha}_{\boldsymbol{k}}$ | Fertility rate for each region *k* relative to the nation’s mean. |  | ^57,58^‬‬‬‬‬‬‬‬‬‬‬‬‬‬‬‬‬‬‬‬‬‬‬‬‬‬‬‬‬‬‬‬‬‬‬‬‬‬‬‬‬‬‬‬‬‬‬‬‬‬‬‬‬‬‬‬‬‬‬‬‬‬‬‬‬‬‬‬‬‬‬‬‬‬‬‬‬‬‬‬‬‬‬‬‬‬‬‬‬‬‬‬‬‬‬‬‬‬‬‬‬‬‬‬‬‬‬‬ |
| $\boldsymbol{\rho}_{\boldsymbol{j}\boldsymbol{,}\boldsymbol{r}}$ | Probability of hospitalization for each age-and risk-group given a reported case | Table S2 | ^31,59^ |

1. **Calibrated parameters**

To estimate empirically unknown epidemiological parameters, we calibrated our model to daily age-stratified cases of COVID-19 confirmed by PCR tests in 30 subdistricts covering Israel between May 15, 2020, and October 25, 2021. We shifted the model 12 days backward, to compensate for the lag between the date of infection and the date of the first positive SARS-CoV2 test result, which was found to be 11.5 days on average according to MOH’s epidemiological investigations. We applied a central moving average with a window of three days before and after the data point, on the data to reduce noise caused by weekly patterns.

The calibration was conducted on a 30-subdistrict level to ensure there are sufficient time-series data points in each location for each age group. The stratification is based on the 16 formal districts, which we further stratified such that the sub-districts will be homogenous in terms of their SES and religious affiliation (Table S4). To calibrate the model to the incidence data, we maximized the likelihood by assuming a normal distribution of the error between model predictions and incidence data. This was achieved by using the truncated Newton (TNC) algorithm. We calibrated the model for 3 different scenarios of unreported cases (see section 2.3, *Unreported cases*). The final transmission model included five parameters without constraints imposed from previous data: intermittent school closures $\hat{\beta}_{school}$, intermittent businesses closure, isolation provision, and populations’ adherence to restrictions $\hat{\beta}_{workplace}$ and susceptibility rate based on age-groups *j*: 0-19, 20-59, and >60 (Table S5).

**Table S4**. **30 subdistricts calibrated.**

| Sub-district number | Name | Population Size |
| --- | --- | --- |
| 1 | Jerusalem and sub. | 778,503 |
| 2 | Bet Shemesh | 120,164 |
| 3 | Jerusalem and sub. (Orthodox Jewish) | 265,313 |
| 4 | Zefat | 138,618 |
| 5 | Zefat (Israeli Arabs) | 23,772 |
| 6 | Kinneret (Jewish) | 98,178 |
| 7 | Jezreel Valley (Israeli Arabs) | 159,112 |
| 8 | Jezreel Valley (Jewish) | 351,446 |
| 9 | Akko (Israeli Arabs) | 357,341 |
| 10 | Akko (Jewish) | 314,607 |
| 11 | Ramat Hagolan | 51,980 |
| 12 | Haifa (Israeli Arabs) | 35,637 |
| 13 | Haifa (Jewish) | 589,951 |
| 14 | Hadera (Israeli Arabs) | 115,000 |
| 15 | Hadera (Jewish) | 315,593 |
| 16 | Sharon (Israeli Arabs) | 85,729 |
| 17 | Sharon (Jewish) | 412,638 |
| 18 | Petah Tiqwa (Israeli Arabs) | 27,455 |
| 19 | Petah Tiqwa (Orthodox Jewish) | 49,549 |
| 20 | Petah Tiqwa (Secular Jewish) | 680,836 |
| 21 | Ramla | 323,352 |
| 22 | Rehovot | 661,079 |
| 23 | Tel Aviv – Yafo | 820,271 |
| 24 | Bnei Brak | 211,259 |
| 25 | Tel Aviv suburbs | 464,974 |
| 26 | Ashqelon | 559,556 |
| 27 | Beer Sheva (Israeli Arabs) | 196,311 |
| 28 | Beer Sheva (Jewish) | 504,831 |
| 29 | Judea and Samaria | 267,832 |
| 30 | Judea and Samaria (Orthodox Jewish) | 155,095 |

**Table S5**. **Calibrated parameters.**

| Reported:unreportd ratio | | Susceptibility among age-group 0-19[y]  $\boldsymbol{\beta}_{\boldsymbol{0}\boldsymbol{-}\boldsymbol{19}}$ | Susceptibility among age-group 20-39[y]  $\boldsymbol{\beta}_{\boldsymbol{20}\boldsymbol{-}\boldsymbol{59}}$ | Susceptibility among age-group 60+[y]  $\boldsymbol{\beta}_{\boldsymbol{60}\boldsymbol{+}}$ | Infectious rate caused by school openings$\boldsymbol{\beta}_{\boldsymbol{school}}$ | Infectious rate caused by economy activity$\boldsymbol{\beta}_{\boldsymbol{workplace}}$ | | | Likelihood of calibration to data  $\boldsymbol{-}\log\boldsymbol{(l)}$ |  |
| --- | --- | --- | --- | --- | --- | --- | --- | --- | --- | --- |
| 1:1 | 0.038 | | 0.037 | 0.088 | 0.500 | | 1.340 | -20.440 | | |
| 1:2 | 0.037 | | 0.038 | 0.082 | 0.540 | | 1.476 | -20.587 | | |
| 1:3 | 0.046 | | 0.036 | 0.099 | 0.566 | | 1.624 | -20.731 | | |

1. **Further results**
   1. **Model calibration**


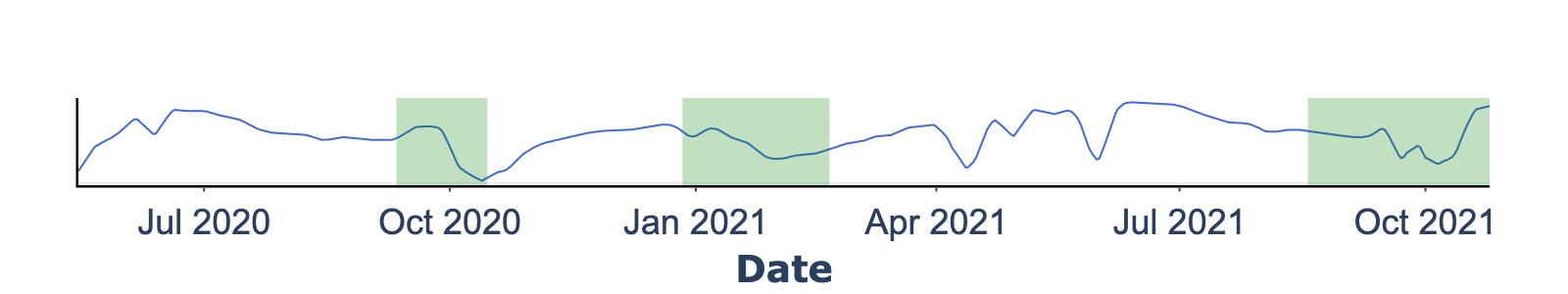

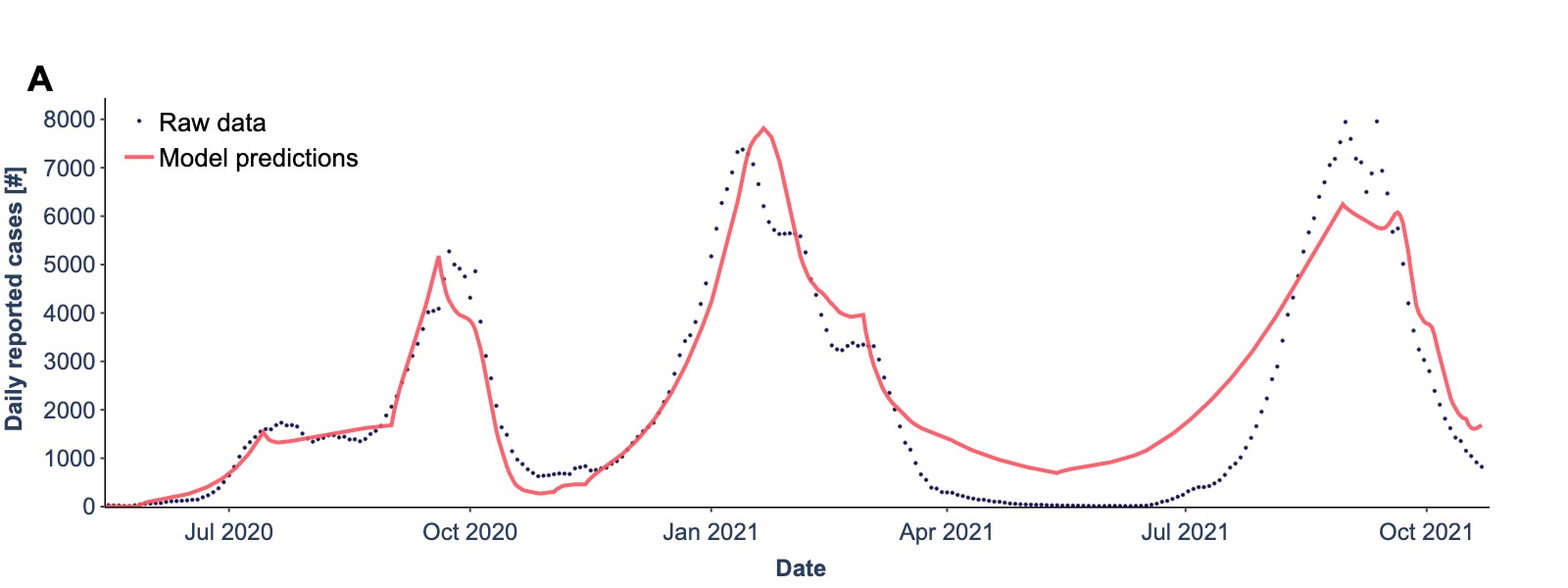


**Fig. S2**. **Fit of the transmission mode, considering three unreported cases for a single reported case**. (**a**) Time series of total daily reported cases of COVID-19 countrywide. We performed the model fit and validation utilizing the total daily reported cases and included a descriptive visualization of the time periods in which the $\hat{\beta}_{workplace}$ influenced the transmission model. (**b**) Data and model fit to the total reported COVID-19 cases among different age groups. (**c**) Data and model fit to the 30 regions covering Israel.


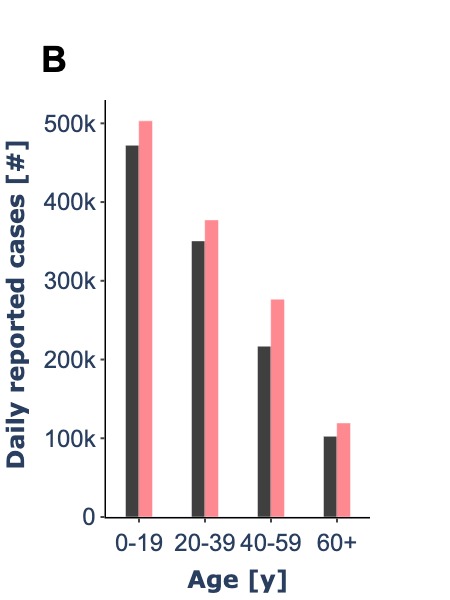

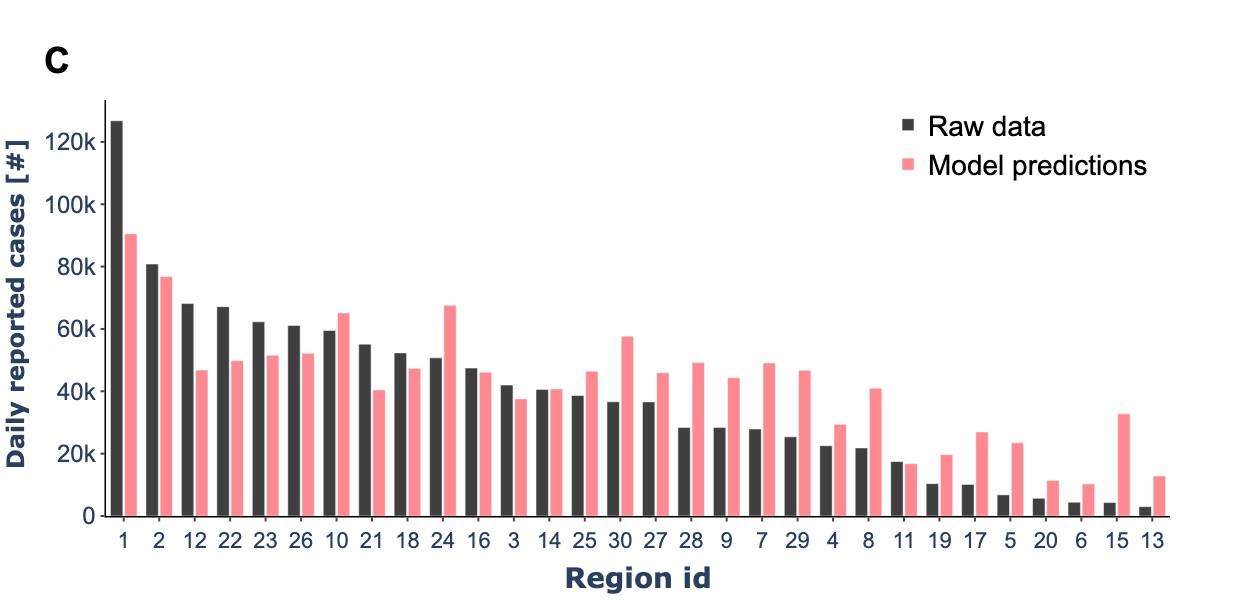

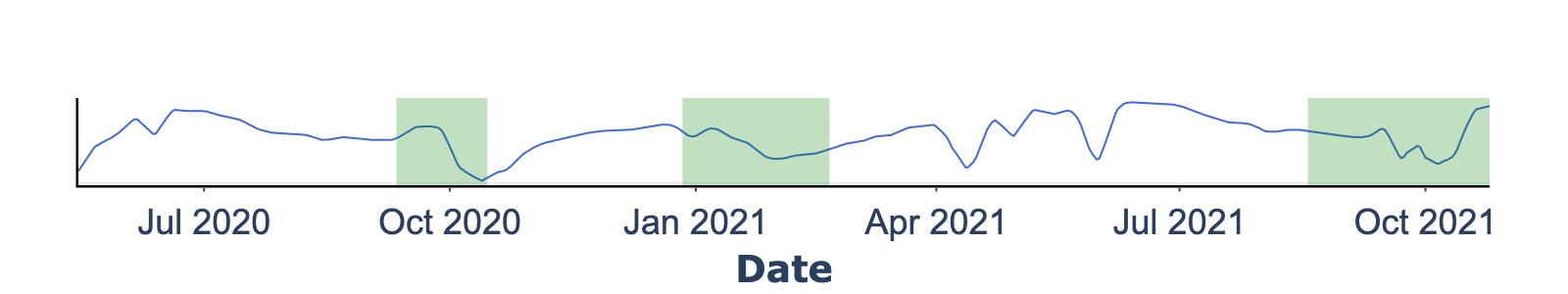

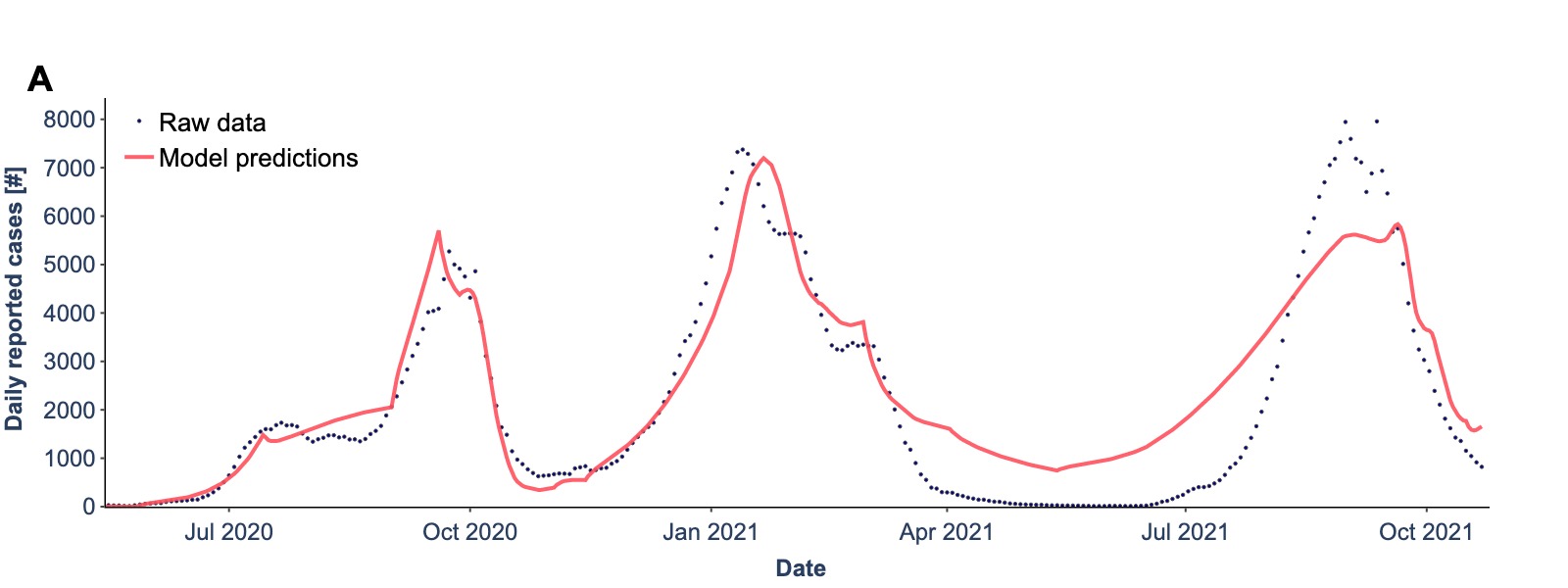

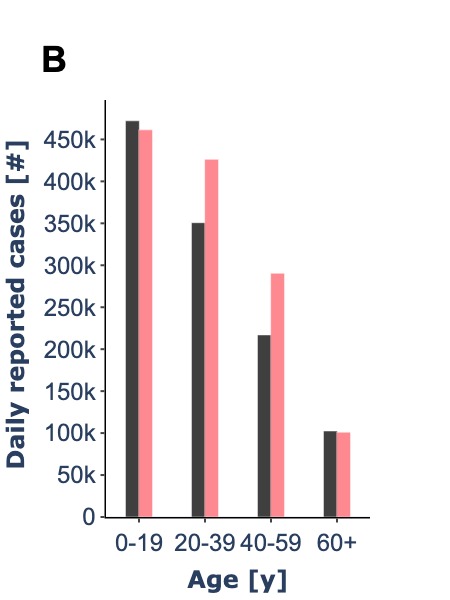


**Fig. S3.** **Fit of the transmission mode, considering two unreported cases for a single reported case**. (**a**) Time series of total daily reported cases of COVID-19 countrywide. We performed the model fit and validation utilizing the total daily reported cases and included a descriptive visualization of the time periods in which the $\hat{\beta}_{workplace}$ influenced the transmission model (**b**) Data and model fit to the total reported COVID-19 cases among different age groups. (**c**) Data and model fit to the 30 regions covering Israel.


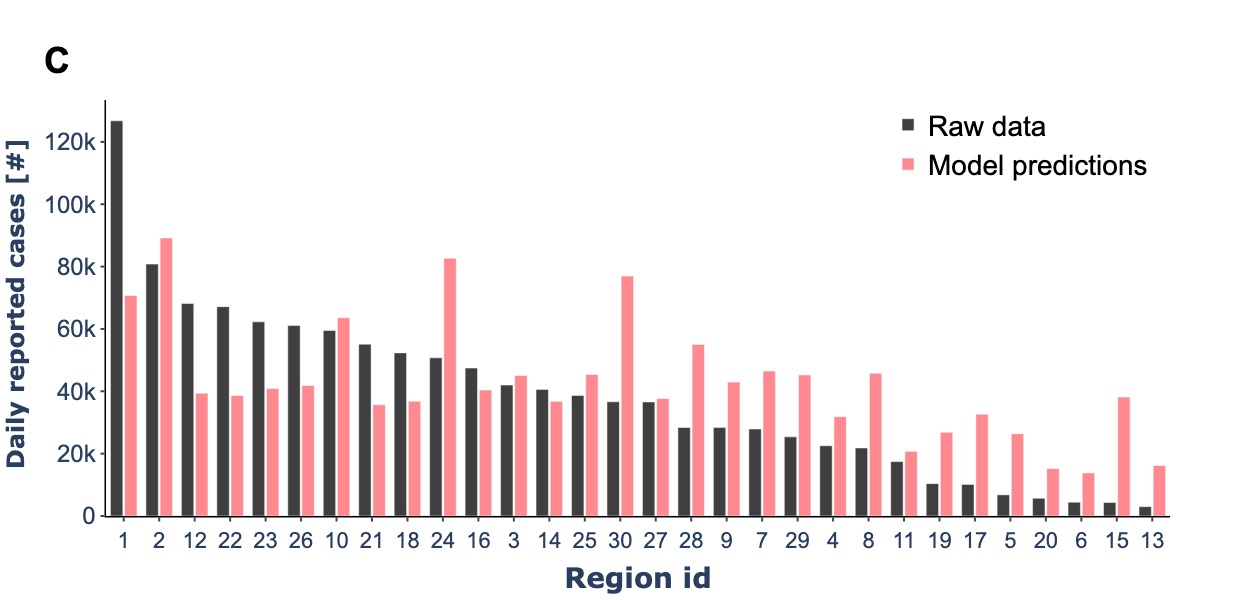

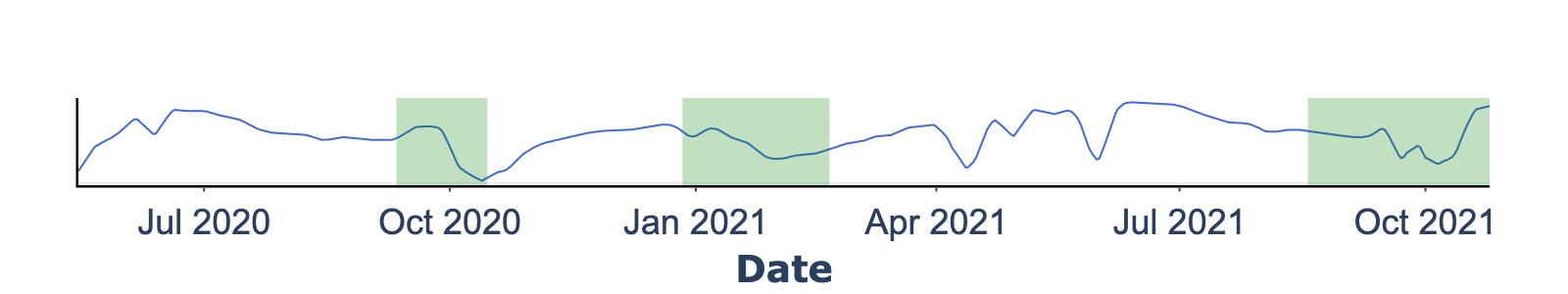

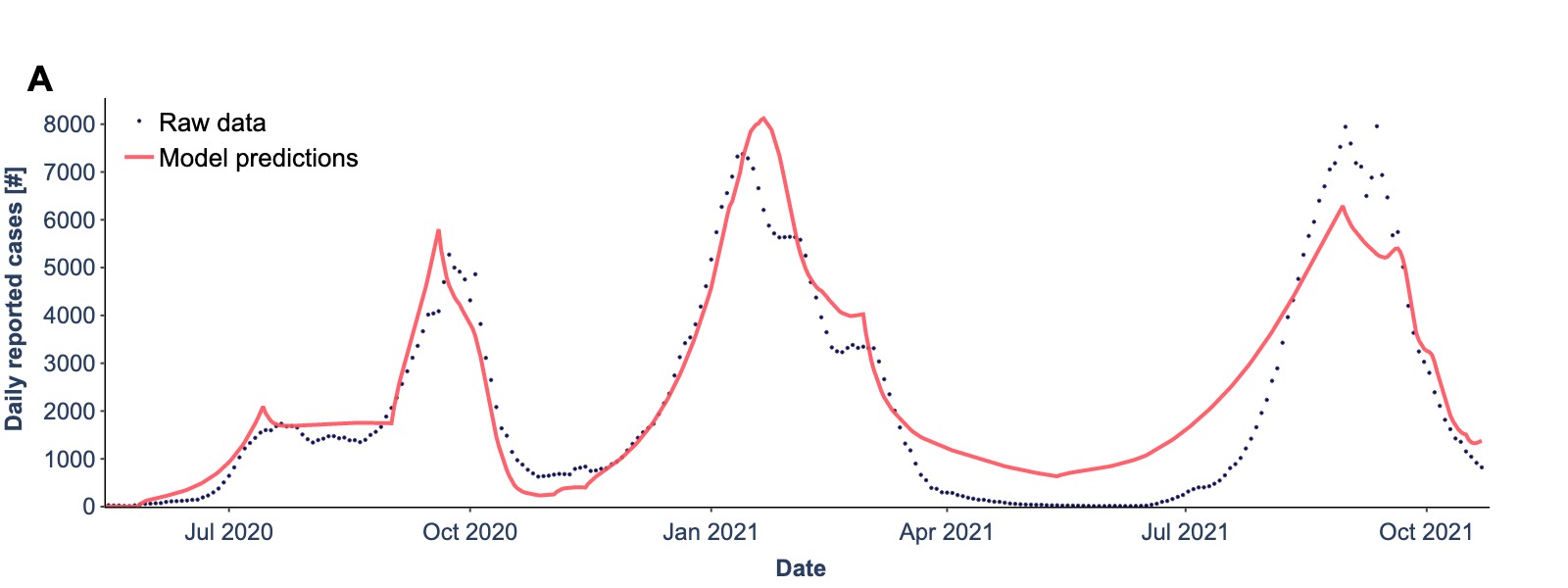


**Fig. S4.** **Fit of the transmission mode, considering one unreported case for a single reported case.** (**a**) Time series of total daily reported cases of COVID-19 countrywide. We performed the model fit and validation utilizing the total daily reported cases and included a descriptive visualization of the time periods in which the $\hat{\beta}_{workplace}$ influenced the transmission model (**b**) Data and model fit to the total reported COVID-19 cases among different age groups. (**c**) Data and model fit to the 30 regions covering Israel.


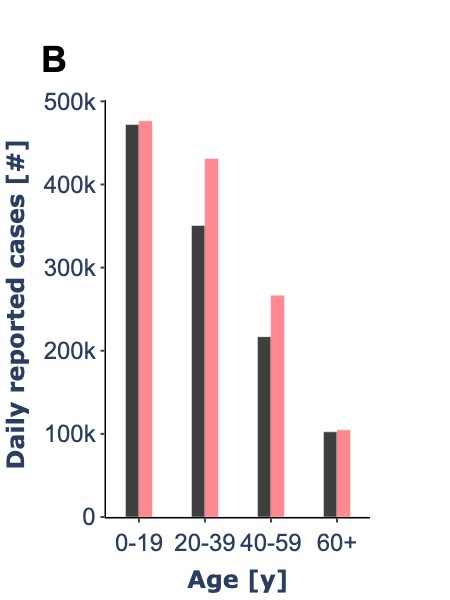

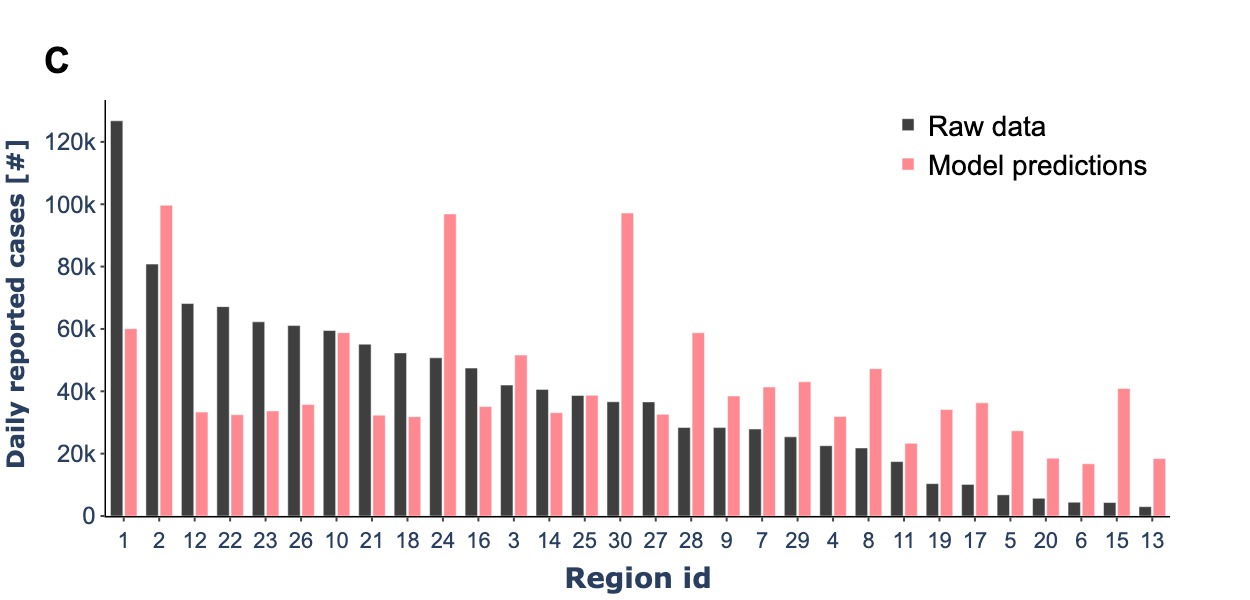


- 1. **Model simulations**


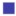
 Morbidity-based, update prioritization monthly


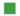
 Morbidity-based, update prioritization every six months


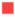
 Mortality-based, update prioritization every six months


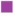
 Mortality-based, update prioritization monthly


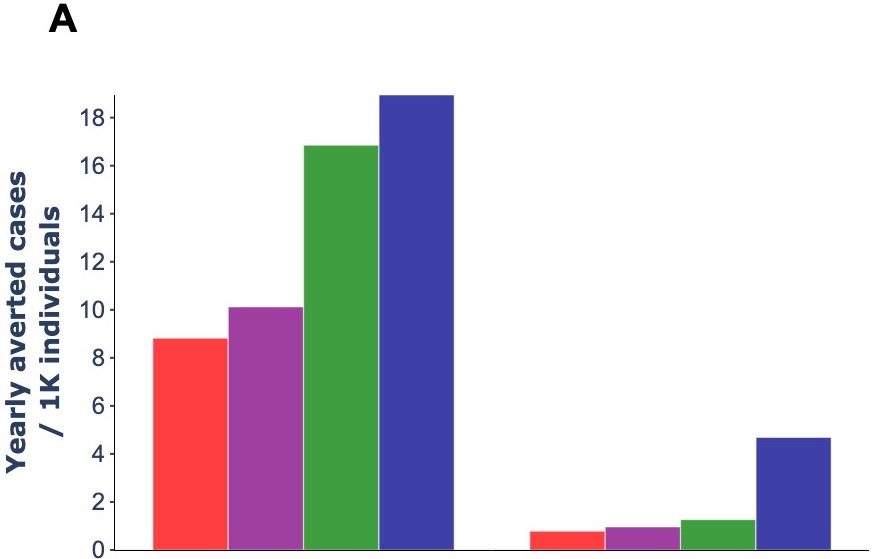

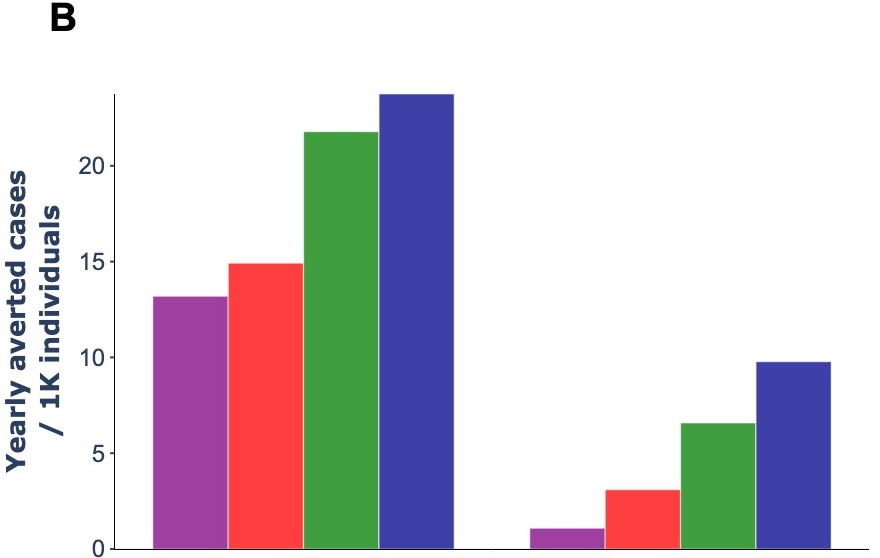

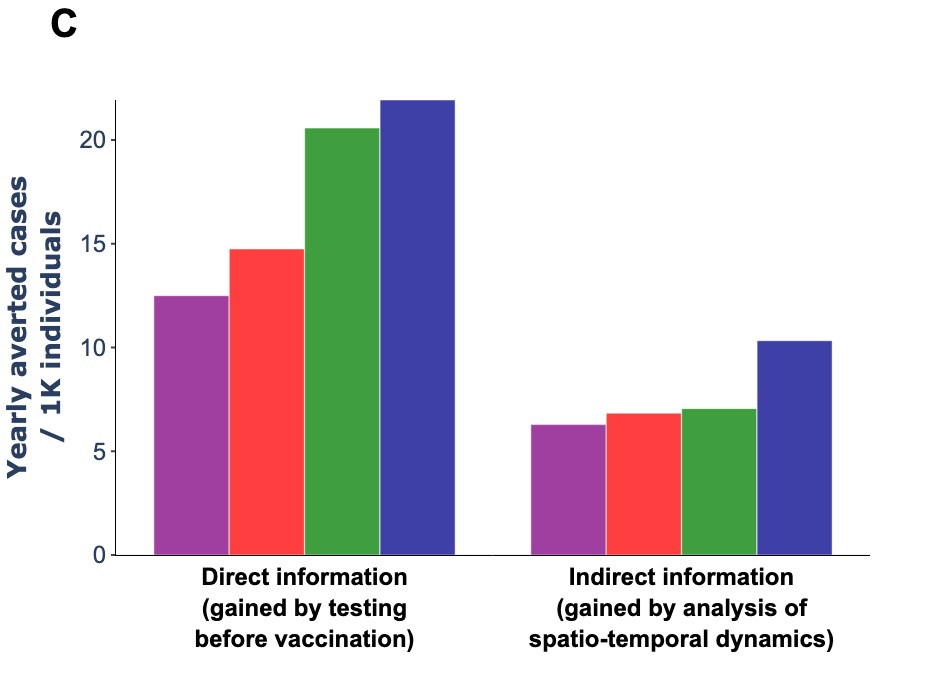


**Fig. S5**. **Effectiveness of vaccination strategies for different inventory levels of vaccinations.** Yearly averted cases (**a, b, c**) per 1,000 individuals for inventory level equals to 10%, 15% and 20% of the entire population (respectively), over time horizon of three years, relative to the baseline strategy and after implementing all suggested strategies. The unreported ratio is three unreported cases for a single reported case.


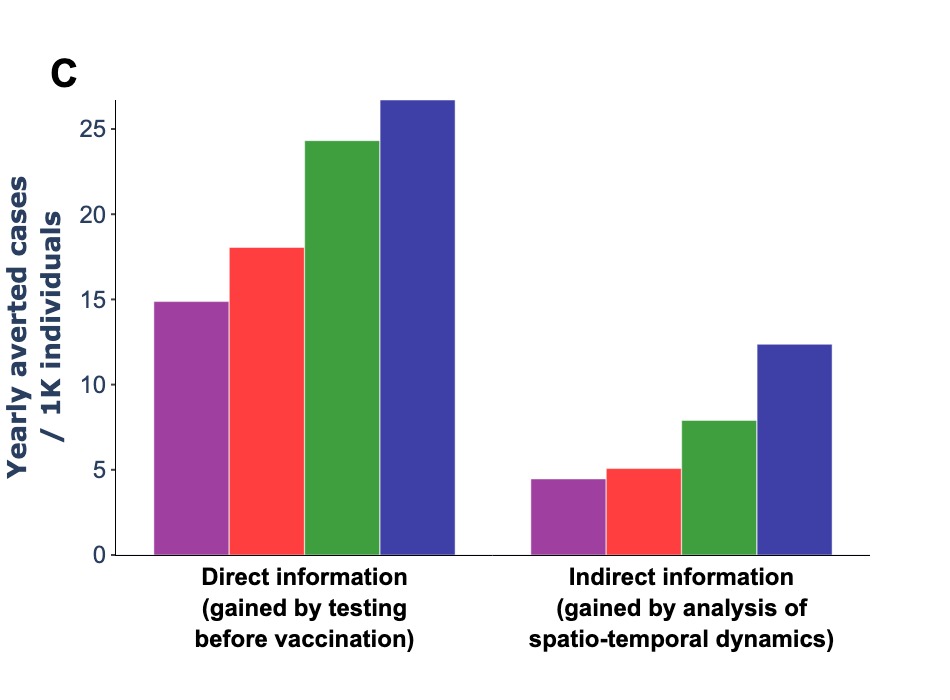

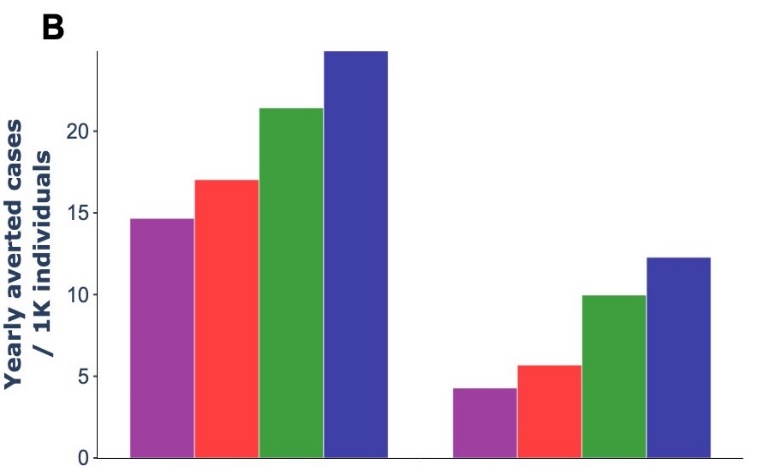

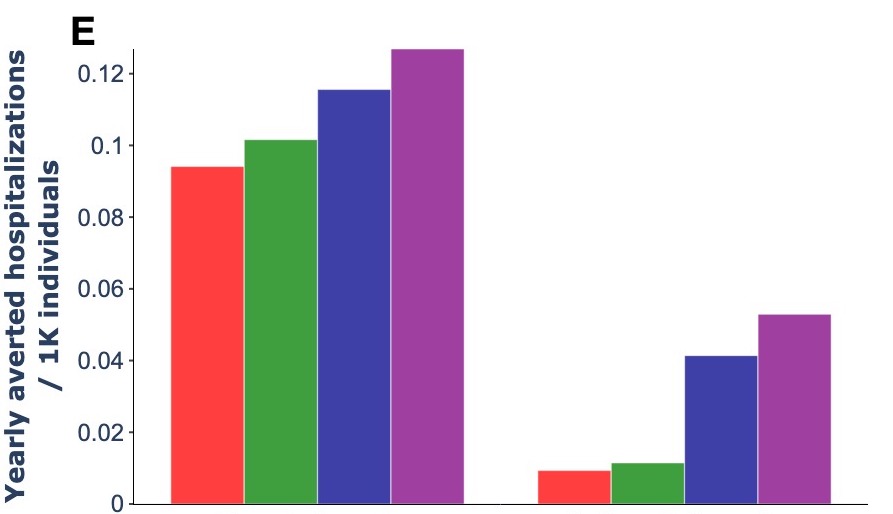

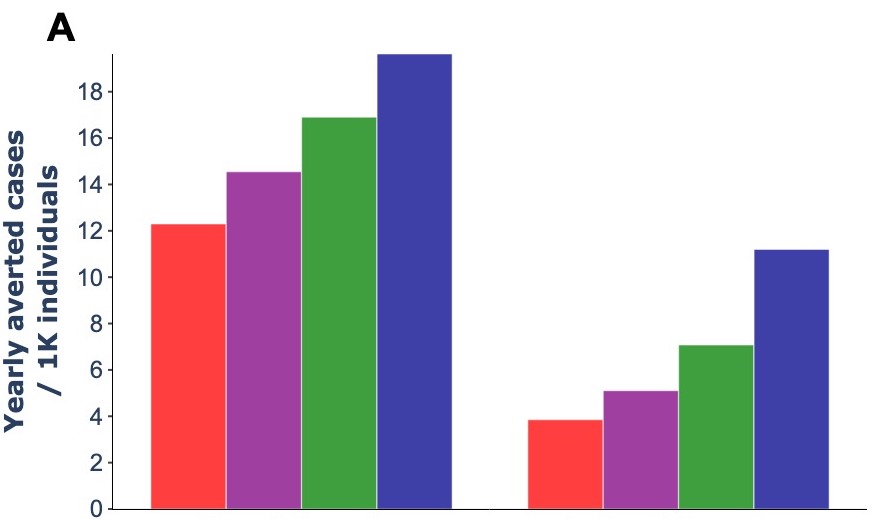

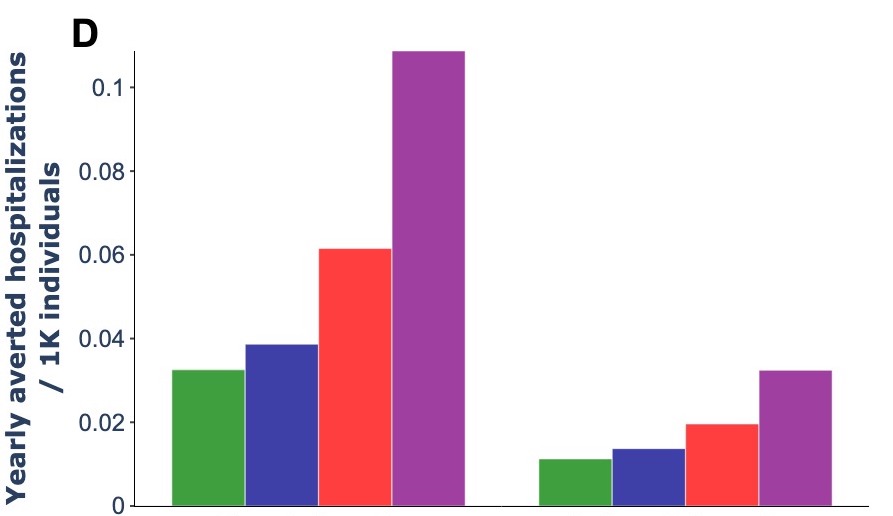

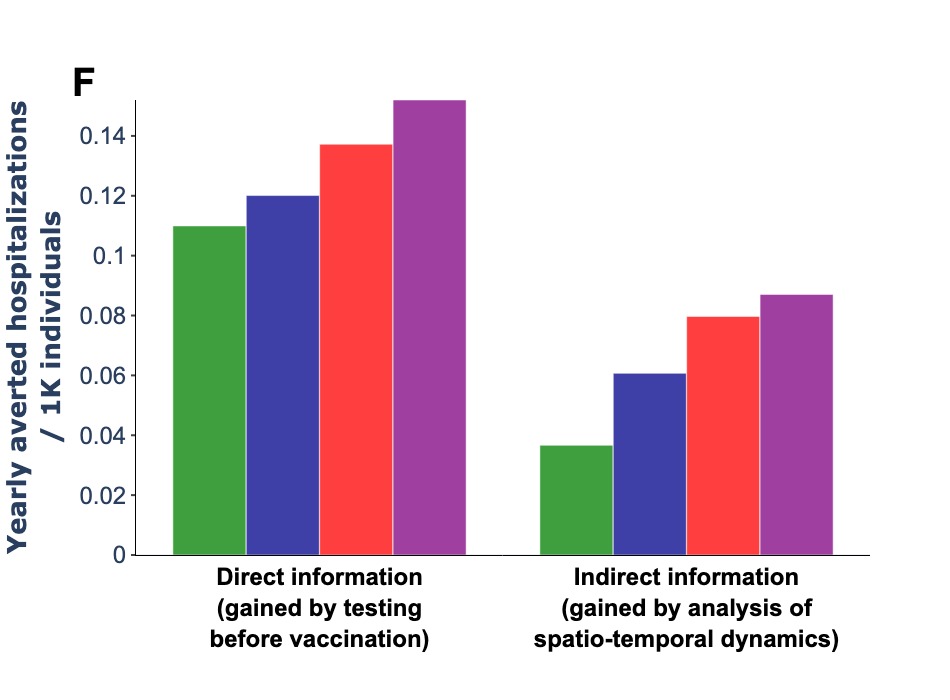

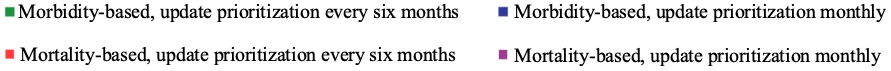


**Fig. S6.** **Effectiveness of vaccination strategies for different inventory levels of vaccinations.** Yearly averted cases (**a, b, c**) and hospitalizations (**d, e, f**) per 1,000 individuals for inventory level equals to 10%, 15% and 20% of the entire population (respectively), over time horizon of three years, relative to the baseline strategy and after implementing all suggested strategies. The unreported ratio is two unreported cases for a single reported case.


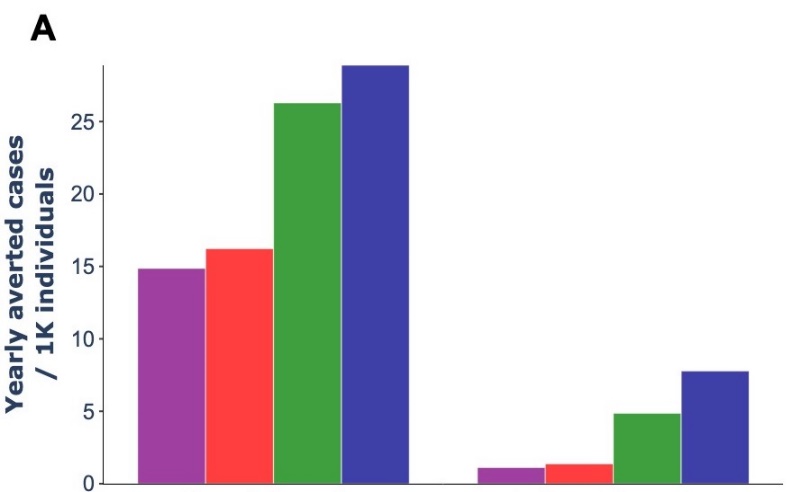

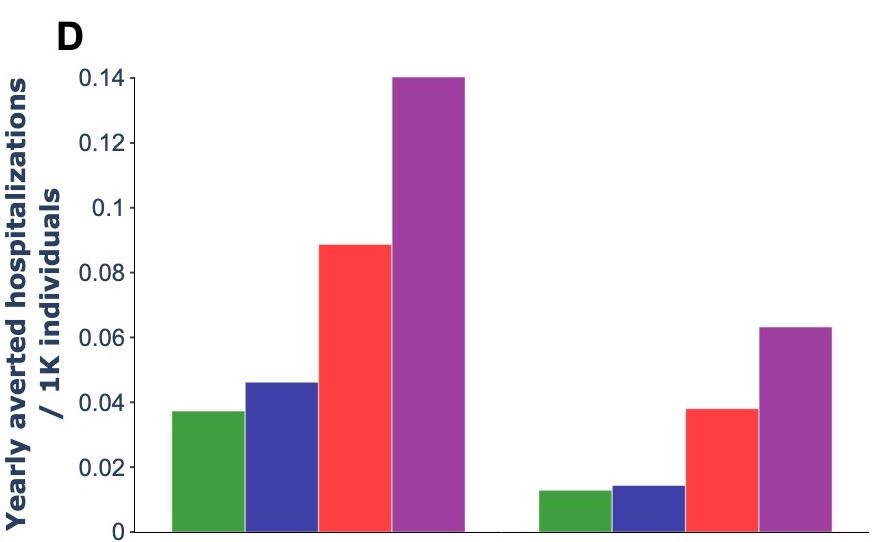

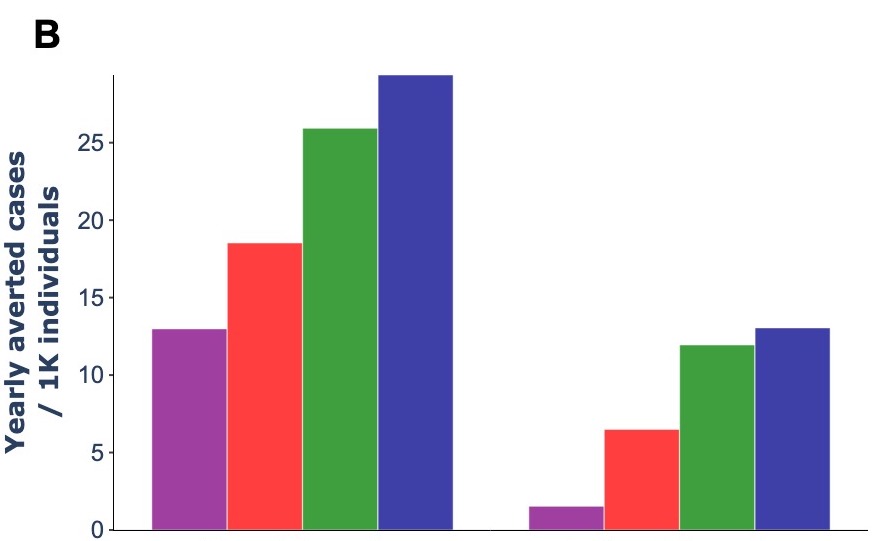

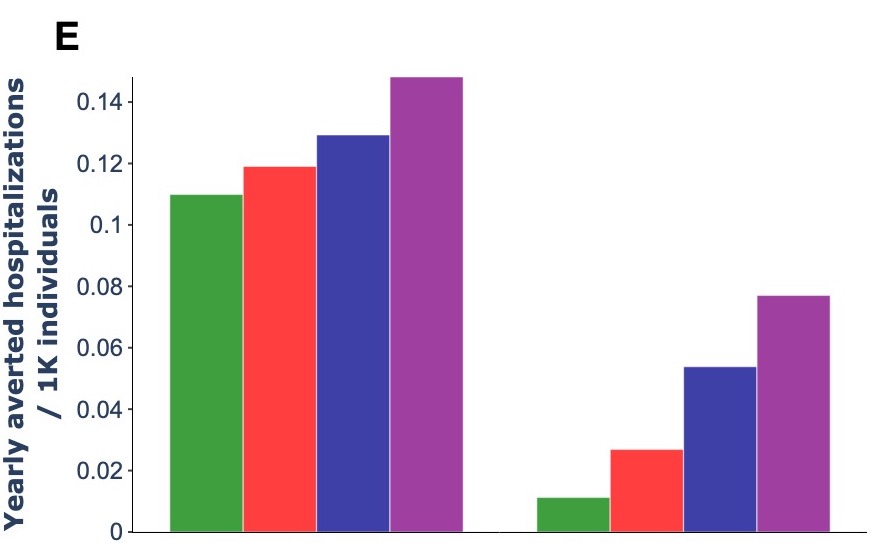

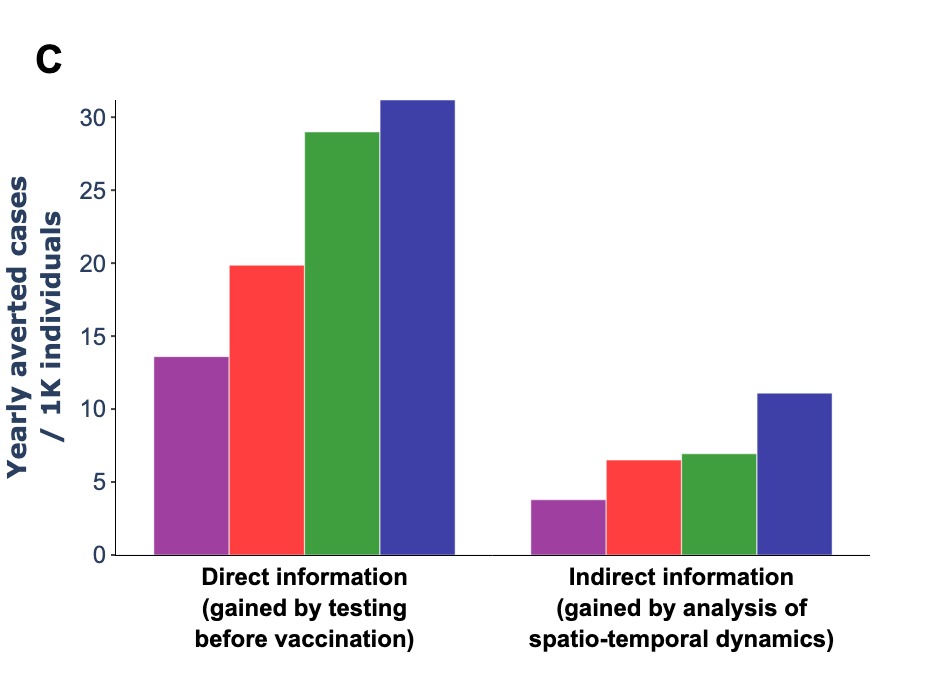

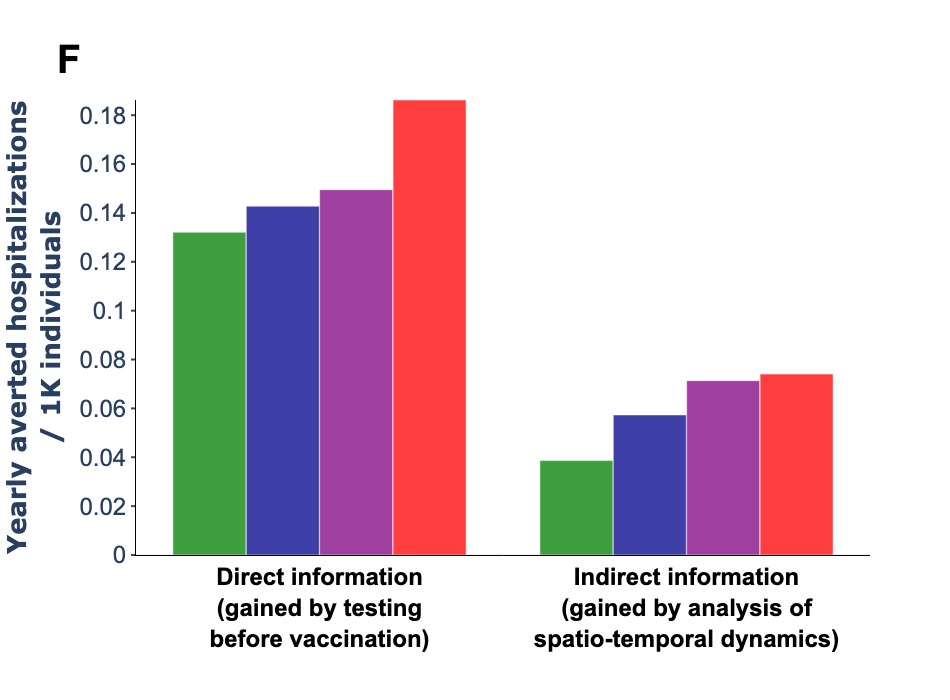

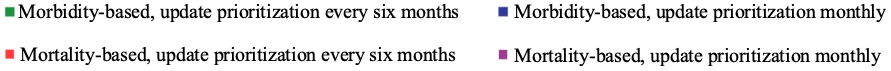


**Fig. S7**. **Effectiveness of vaccination strategies for different inventory levels of vaccinations.** Yearly averted cases (**a, b, c**) and hospitalizations (**d, e, f**) per 1,000 individuals for inventory level equals to 10%, 15% and 20% of the entire population (respectively), over time horizon of three years, relative to the baseline strategy and after implementing all suggested strategies. The unreported ratio is one unreported case for a single reported case.

- 1. **Sensitivity analyses**


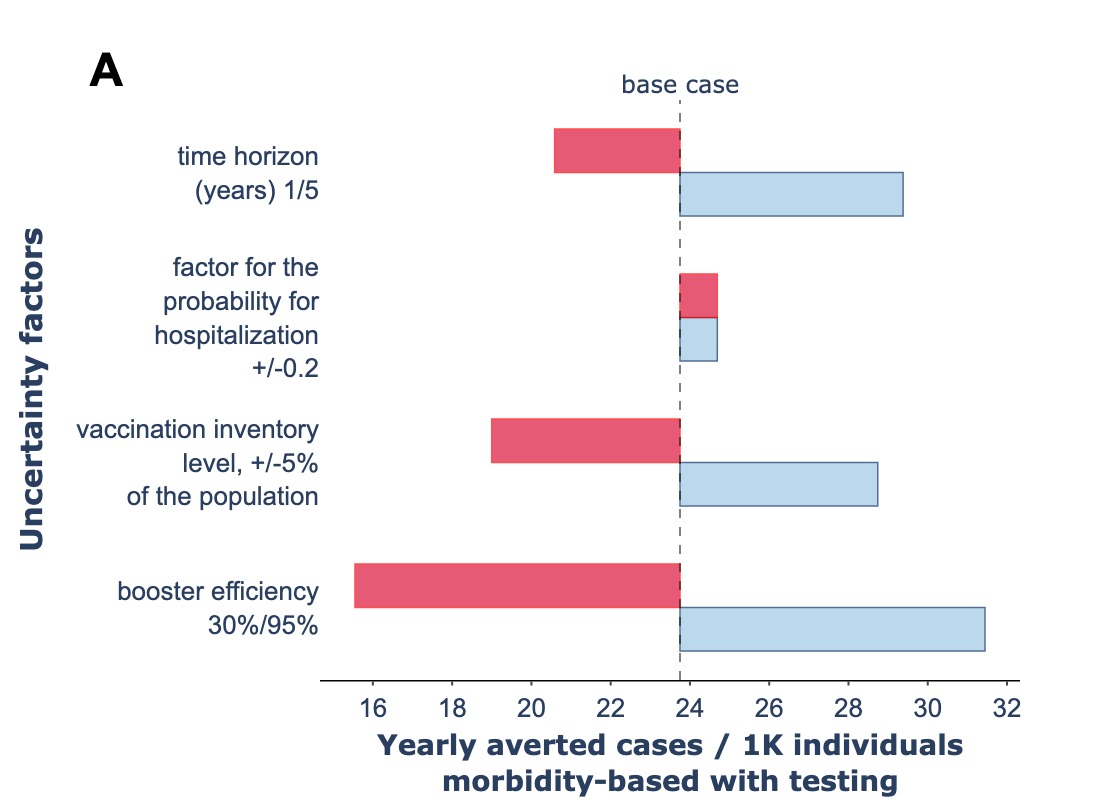

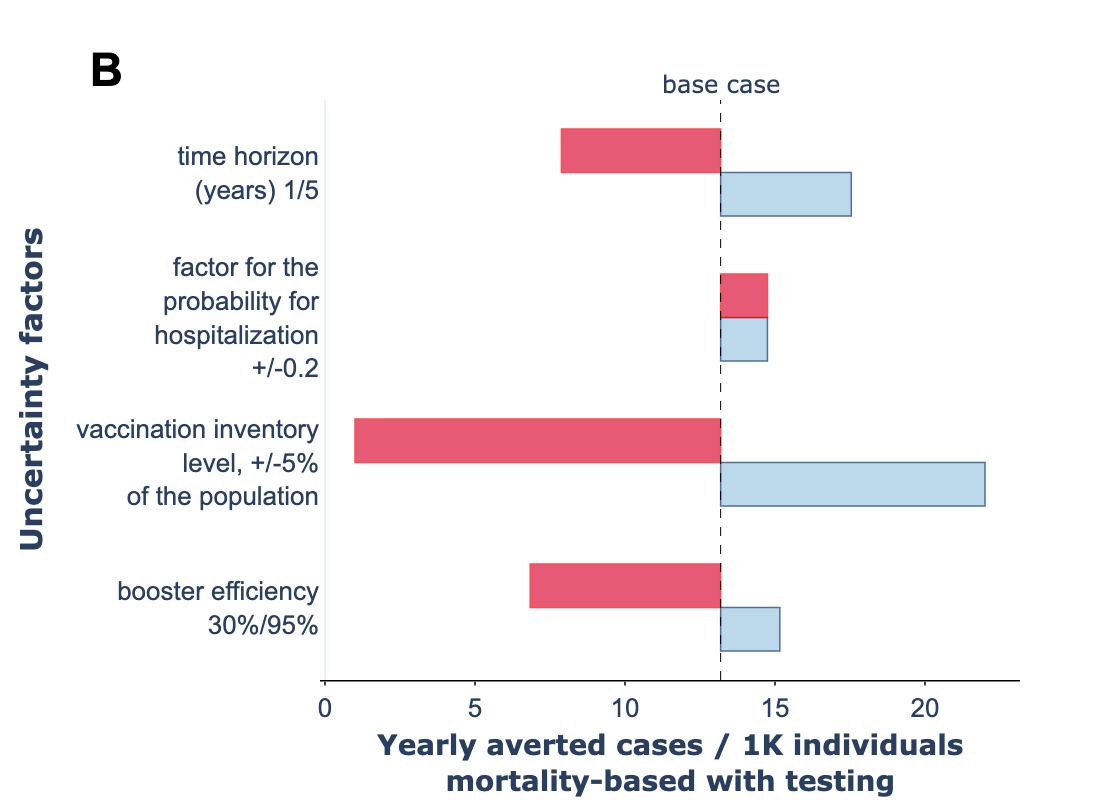


**Fig. 8.** **Univariate sensitivity analysis of the yearly averted reported cases (a, b) per one-thousand individuals, relative to the baseline.** The unreported ratio is one unreported case for a single reported case.


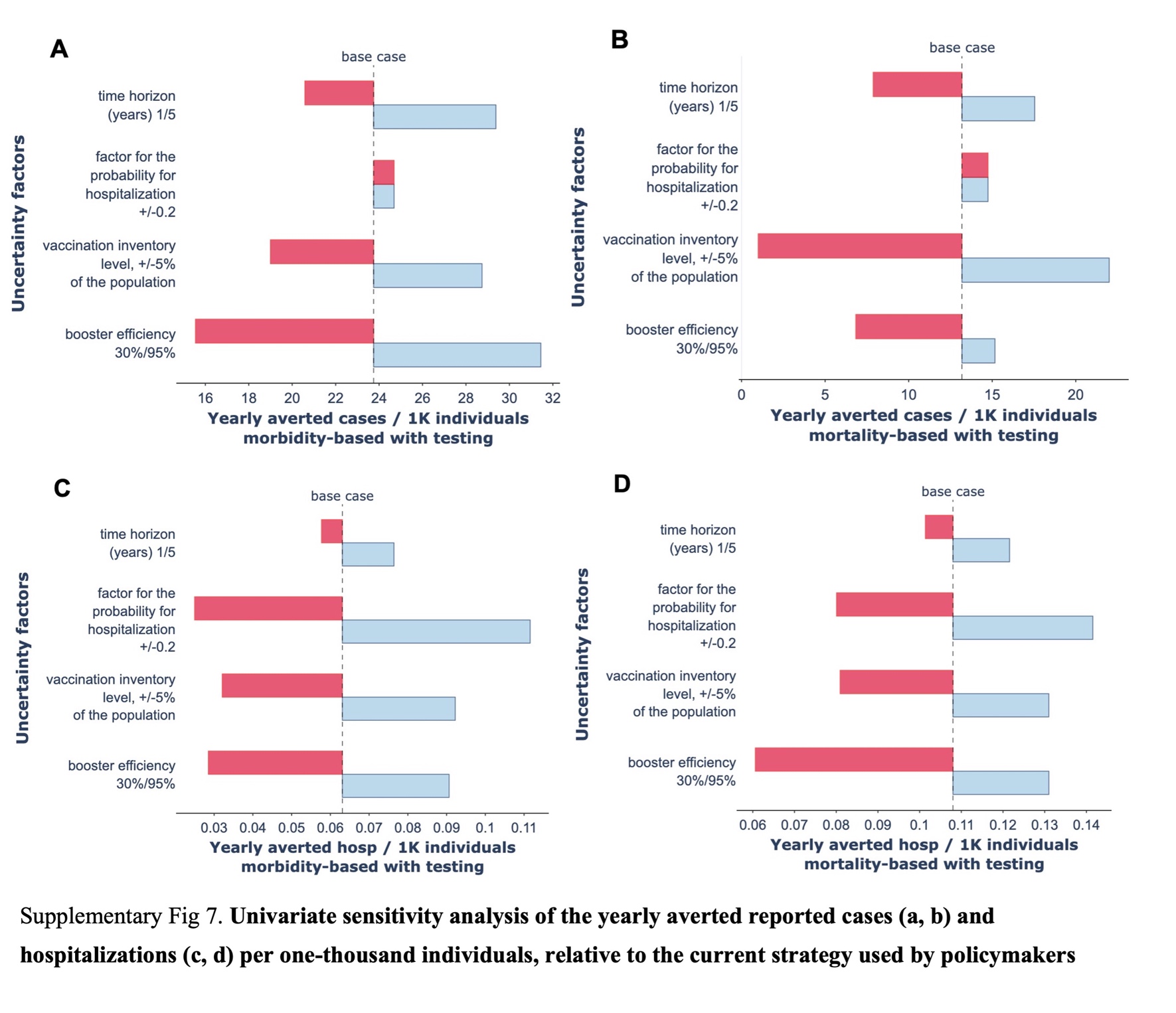


**Fig. S9.** **Univariate sensitivity analysis of the yearly averted reported cases (a, b) and hospitalizations (c, d) per one-thousand individuals, relative to the baseline strategy.** The unreported ratio is two unreported cases for a single reported case.


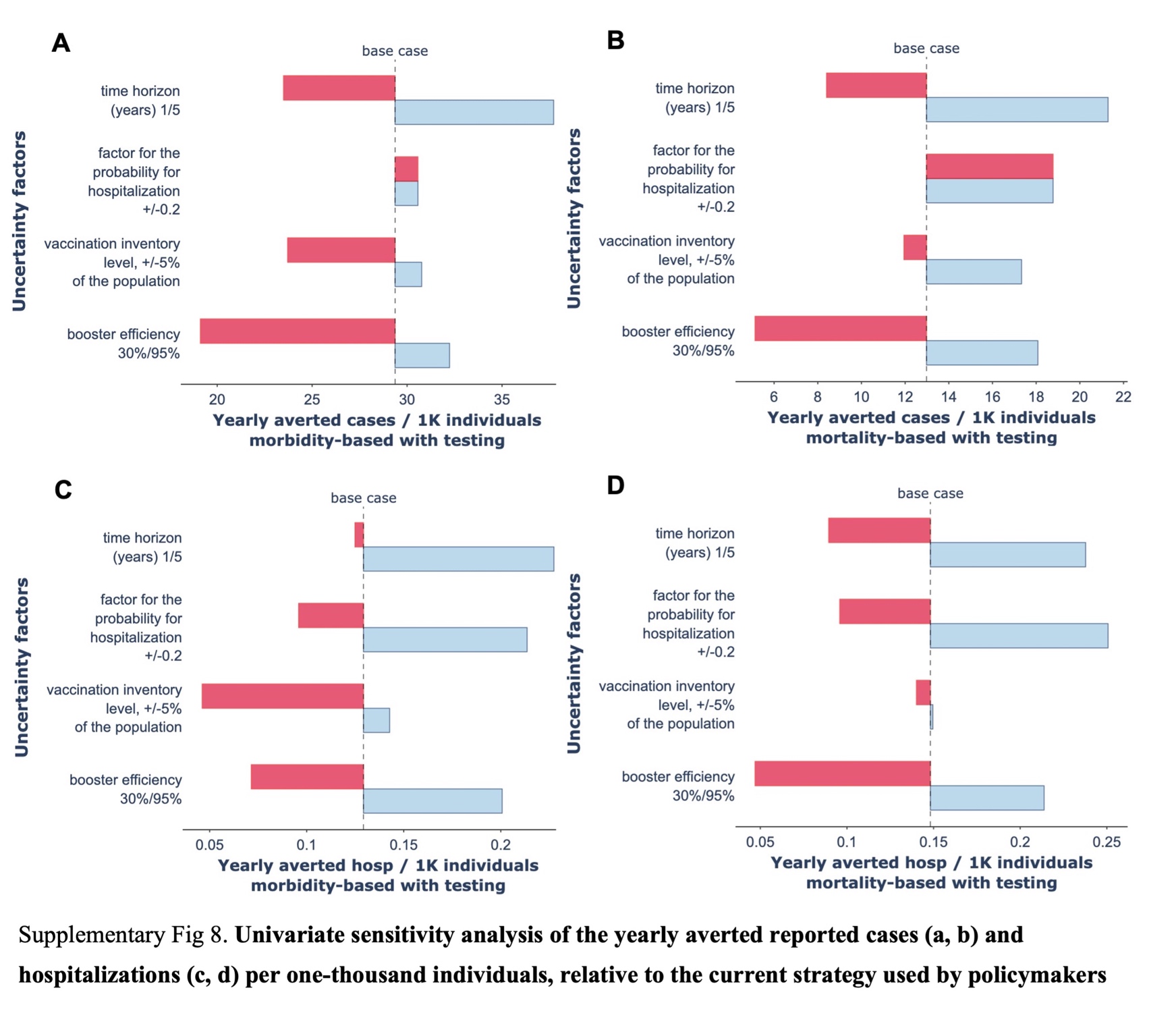


**Fig. S10.** **Univariate sensitivity analysis of the yearly averted reported cases (a, b) and hospitalizations (c, d) per one-thousand individuals, relative to the baseline strategy.** The unreported ratio is one unreported case for a single reported case.


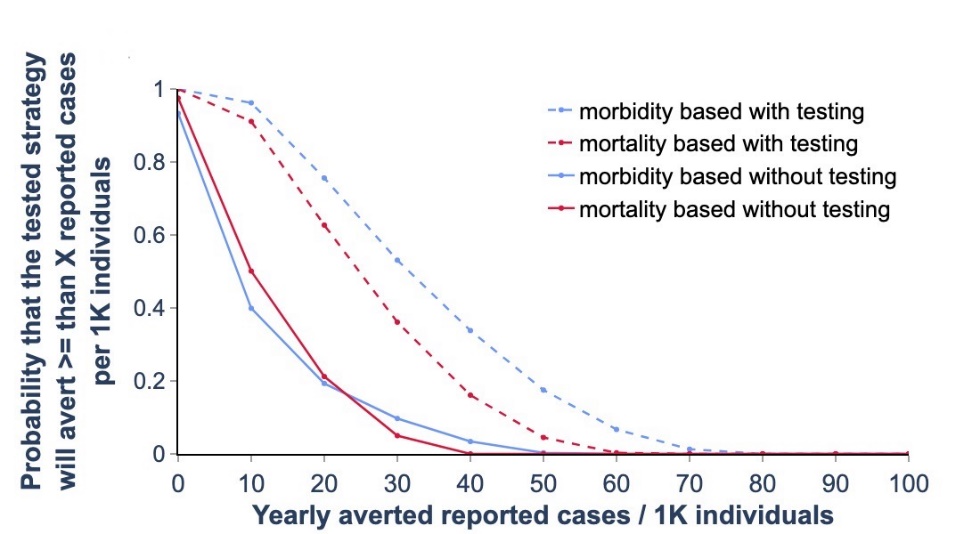


**Fig. S11 Global uncertainty analysis results.** The threshold curves show the effect of a suggested strategy on the probability to avert an equal amount of reported cases per one-thousand individuals per year, or higher, relative to the baseline strategy. The unreported ratio is three unreported cases per reported case.

**Fig. S12.** **Global uncertainty analysis results.** Threshold curves showing the effect of a suggested strategy on the probability to avert an equal amount of the yearly reported cases (**a**) and hospitalizations (**b**) per one-thousand individuals, relative to the baseline strategy. The unreported ratio is two unreported cases for a single reported case.


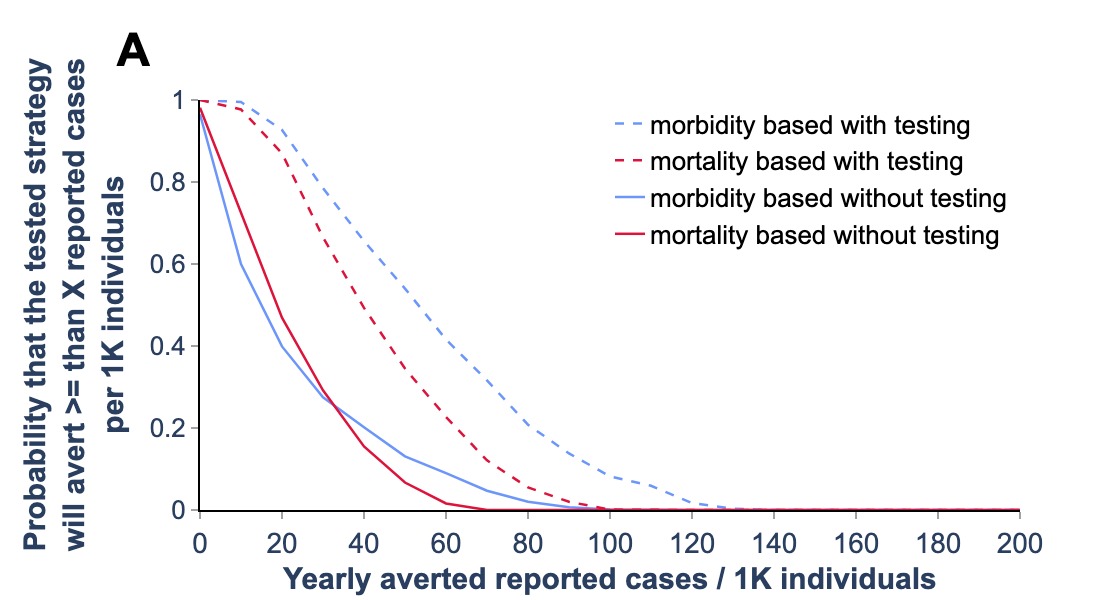

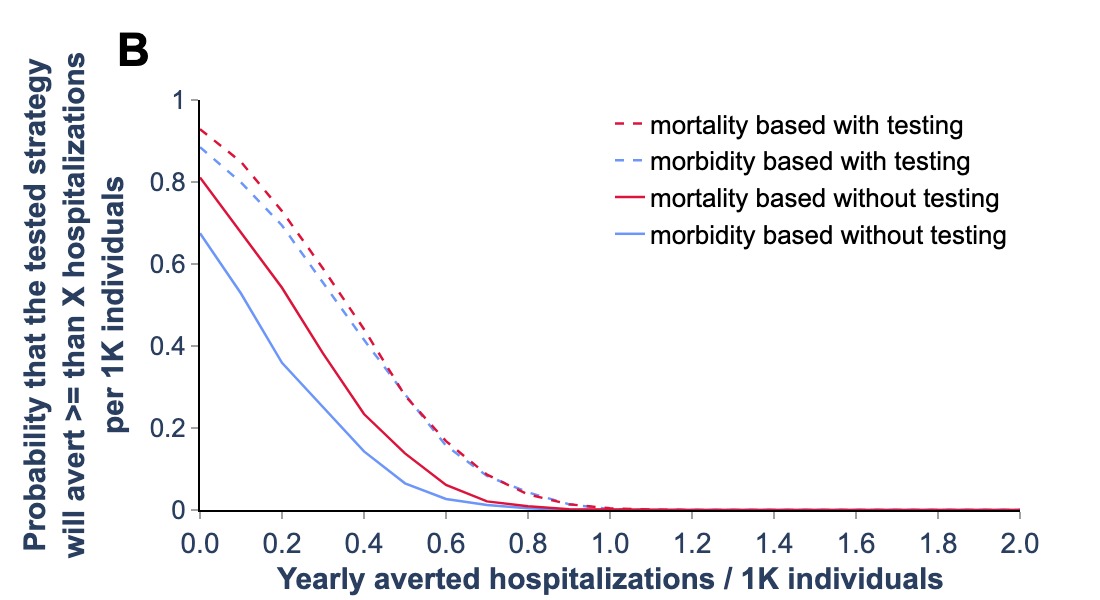


**Fig. S13.** **Global uncertainty analysis results.** Threshold curves showing the effect of a suggested strategy on the probability to avert an equal amount of the yearly reported cases (**a**) and hospitalizations (**b**) per one-thousand individuals, relative to the baseline strategy. The unreported ratio is one unreported case for a single reported case.


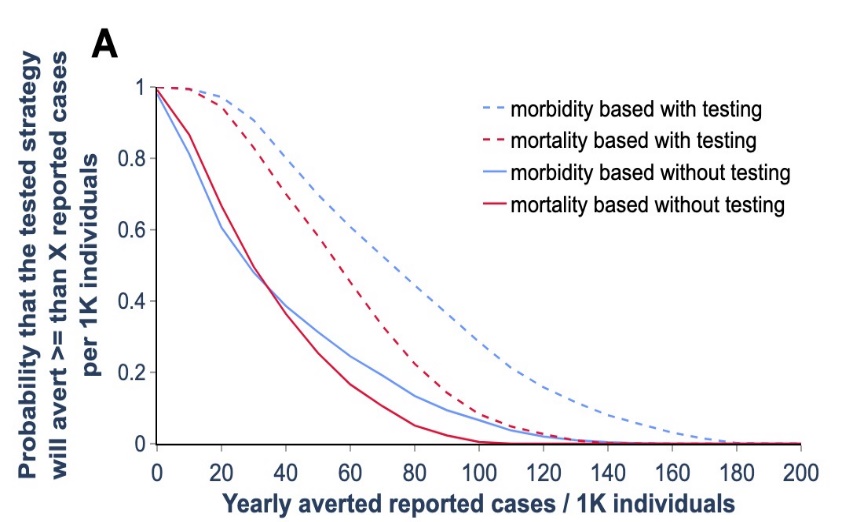

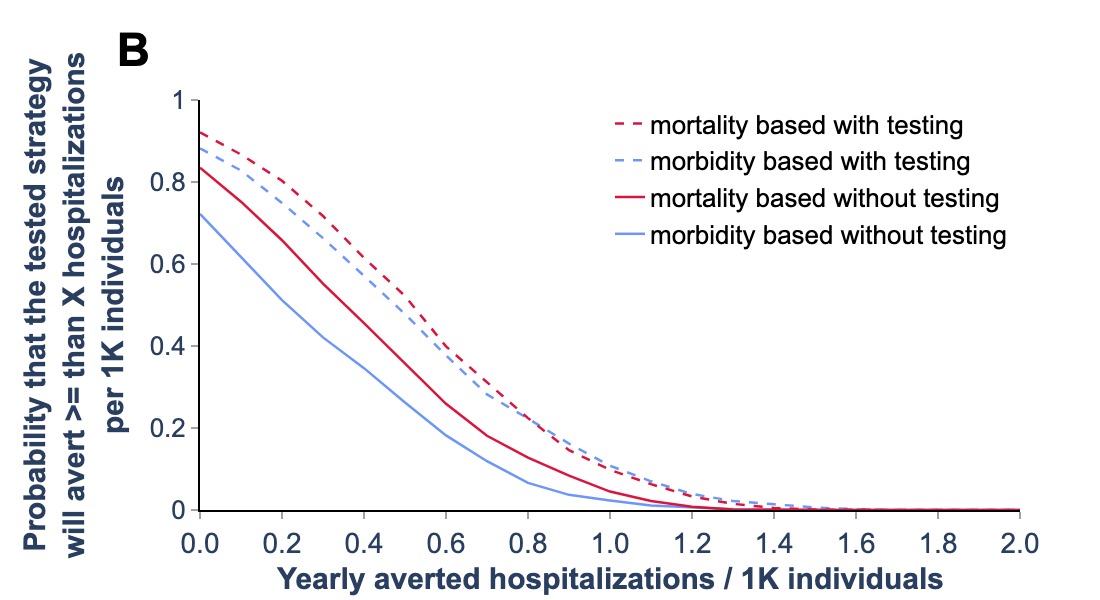


1. **References**

1. Vynnycky, E. & White, R. Introduction. The basics: infections, transmission and models. in *An Introduction to Infectious Disease Modelling* (2010).

2. Medlock, J. & Galvani, A. P. Optimizing influenza vaccine distribution. *Science (1979)* (2009) doi:10.1126/science.1175570.

3. Ndeffo Mbah, M. L., Medlock, J., Meyers, L. A., Galvani, A. P. & Townsend, J. P. Optimal targeting of seasonal influenza vaccination toward younger ages is robust to parameter uncertainty. *Vaccine* (2013) doi:10.1016/j.vaccine.2013.04.052.

4. Yamin, D. *et al.* An Innovative Influenza Vaccination Policy: Targeting Last Season’s Patients. *PLoS Comput Biol* **10**, e1003643 (2014).

5. Molinari, N. A. M. *et al.* The annual impact of seasonal influenza in the US: Measuring disease burden and costs. *Vaccine* (2007) doi:10.1016/j.vaccine.2007.03.046.

6. Fiore, A. E. *et al.* Antiviral agents for the treatment and chemoprophylaxis of influenza --- recommendations of the Advisory Committee on Immunization Practices (ACIP). *MMWR. Surveillance summaries : Morbidity and mortality weekly report. Surveillance summaries / CDC* (2011).

7. Draft landscape and tracker of COVID-19 candidate vaccines. Preprint at https://www.who.int/publications/m/item/draft-landscape-of-covid-19-candidate-vaccines.

8. Munitz, A., Yechezkel, M., Dickstein, Y., Yamin, D. & Gerlic, M. BNT162b2 vaccination effectively prevents the rapid rise of SARS-CoV-2 variant B.1.1.7 in high-risk populations in Israel. *Cell Rep Med* **0**, 100264 (2021).

9. Sharma, O., Sultan, A. A., Ding, H. & Triggle, C. R. A Review of the Progress and Challenges of Developing a Vaccine for COVID-19. *Front Immunol* **11**, 585354 (2020).

10. Parker, E. P. K., Shrotri, M. & Kampmann, B. Keeping track of the SARS-CoV-2 vaccine pipeline. *Nat Rev Immunol* **20**, 650 (2020).

11. Krammer, F. SARS-CoV-2 vaccines in development. *Nature* **586**, 516–527 (2020).

12. Rosen, B., Waitzberg, R. & Israeli, A. Israel’s rapid rollout of vaccinations for COVID-19. *Israel Journal of Health Policy Research 2021 10:1* **10**, 1–14 (2021).

13. Rossman, H. *et al.* COVID-19 dynamics after a national immunization program in Israel. *Nature Medicine 2021 27:6* **27**, 1055–1061 (2021).

14. COVID-19 in Israel dashboard. 2021.

15. Bar-On, Y. M. *et al.* BNT162b2 vaccine booster dose protection: A nationwide study from Israel. *medRxiv* 2021.08.27.21262679 (2021) doi:10.1101/2021.08.27.21262679.

16. Gazit, S. *et al.* Comparing SARS-CoV-2 natural immunity to vaccine-induced immunity: reinfections versus breakthrough infections. doi:10.1101/2021.08.24.21262415.

17. A million Israelis get third dose, with early data showing heightened protection | The Times of Israel. https://www.timesofisrael.com/a-million-israelis-get-third-dose-with-early-data-showing-heightened-protection/.

18. Israel reportedly set to offer COVID boosters to all starting next month | The Times of Israel. https://www.timesofisrael.com/israel-reportedly-set-to-offer-covid-boosters-to-all-starting-next-month/.

19. Khoury, D. S. *et al.* Neutralizing antibody levels are highly predictive of immune protection from symptomatic SARS-CoV-2 infection. *Nature Medicine 2021 27:7* **27**, 1205–1211 (2021).

20. Yamin, D. *et al.* Vaccination strategies against respiratory syncytial virus. *Proc Natl Acad Sci U S A* (2016) doi:10.1073/pnas.1522597113.

21. Sheehan, M. M., Reddy, A. J. & Rothberg, M. B. Reinfection Rates among Patients who Previously Tested Positive for COVID-19: a Retrospective Cohort Study. *medRxiv* 2021.02.14.21251715 (2021) doi:10.1101/2021.02.14.21251715.

22. Past Covid-19 reduces risk of reinfection from Delta variant more than Pfizer vaccine | South China Morning Post. https://www.scmp.com/news/world/middle-east/article/3146656/past-covid-19-reduces-risk-reinfection-delta-variant-pfizer.

23. Stamatatos, L. *et al.* mRNA vaccination boosts cross-variant neutralizing antibodies elicited by SARS-CoV-2 infection. *Science (1979)* **372**, 1413–1418 (2021).

24. Cavanaugh, A. M. Reduced Risk of Reinfection with SARS-CoV-2 After COVID-19 Vaccination — Kentucky, May–June 2021. *MMWR Morb Mortal Wkly Rep* **70**, 1081–1083 (2021).

25. Prior Covid-19 infection reduces infection risk for up to 10 months | UCL News - UCL – University College London. https://www.ucl.ac.uk/news/2021/jun/prior-covid-19-infection-reduces-infection-risk-10-months.

26. Bendavid, E. *et al.* COVID-19 Antibody Seroprevalence in Santa Clara County, California. *medRxiv* 2020.04.14.20062463 (2020) doi:10.1101/2020.04.14.20062463.

27. Gudbjartsson, D. F. *et al.* Spread of SARS-CoV-2 in the Icelandic Population. *New England Journal of Medicine* (2020) doi:10.1056/nejmoa2006100.

28. *(No Title)*.

29. Czech study shows very low COVID-19 incidence in population. https://medicalxpress.com/news/2020-05-czech-covid-incidence-population.html.

30. Kriss, J. L. COVID-19 Vaccine Second-Dose Completion and Interval Between First and Second Doses Among Vaccinated Persons — United States, December 14, 2020−February 14, 2021. *MMWR Morb Mortal Wkly Rep* **70**, 389–395 (2021).

31. Yechezkel, M. *et al.* Human mobility and poverty as key drivers of COVID-19 transmission and control. *BMC Public Health* **21**, 1–13 (2021).

32. COVID-19: Google Mobility Trends - Our World in Data. https://ourworldindata.org/covid-google-mobility-trends.

33. מאגר COVID-19 - נתוני קורונה איזורים סטטיסטיים covid-19 by area - Government Data. https://data.gov.il/dataset/covid-19/resource/d07c0771-01a8-43b2-96cc-c6154e7fa9bd.

34. מאגר COVID-19 - מתחסנים על פי ישוב - Government Data. https://data.gov.il/dataset/covid-19/resource/12c9045c-1bf4-478a-a9e1-1e876cc2e182.

35. מאגר COVID-19 - נתוני קורונה קבוצות מין וגיל - Government Data. https://data.gov.il/dataset/covid-19/resource/89f61e3a-4866-4bbf-bcc1-9734e5fee58e.

36. מאגר COVID-19 - נתוני קורונה בידודים - Government Data. https://data.gov.il/dataset/covid-19/resource/9eedd26c-019b-433a-b28b-efcc98de378d.

37. Zhou, F. *et al.* Clinical course and risk factors for mortality of adult inpatients with COVID-19 in Wuhan, China: a retrospective cohort study. *The Lancet* **395**, 1054–1062 (2020).

38. Coronavirus (COVID-19) Testing - Our World in Data. https://ourworldindata.org/coronavirus-testing.

39. Ma, Q. *et al.* Global Percentage of Asymptomatic SARS-CoV-2 Infections Among the Tested Population and Individuals With Confirmed COVID-19 Diagnosis: A Systematic Review and Meta-analysis. *JAMA Netw Open* **4**, e2137257–e2137257 (2021).

40. Aronna, M. S., Guglielmi, R. & Moschen, L. M. Estimate of the rate of unreported COVID-19 cases during the first outbreak in Rio de Janeiro. *Infect Dis Model* **7**, 317–332 (2022).

41. Estimated COVID-19 Burden | CDC. https://www.cdc.gov/coronavirus/2019-ncov/cases-updates/burden.html.

42. Tracking SARS-CoV-2 variants. https://www.who.int/en/activities/tracking-SARS-CoV-2-variants/.

43. Galloway, S. E. Emergence of SARS-CoV-2 B.1.1.7 Lineage — United States, December 29, 2020–January 12, 2021. *MMWR Morb Mortal Wkly Rep* **70**, 95–99 (2021).

44. Covid: Charts show how far delta variant has spread around the world. https://www.cnbc.com/2021/08/06/covid-charts-show-how-far-delta-variant-has-spread-around-the-world.html.

45. Munitz, A., Yechezkel, M., Dickstein, Y., Yamin, D. & Gerlic, M. BNT162b2 vaccination effectively prevents the rapid rise of SARS-CoV-2 variant B.1.1.7 in high-risk populations in Israel. *Cell Rep Med* **2**, 100264 (2021).

46. Omicron, Delta, Alpha, and More: What To Know About the Coronavirus Variants > News > Yale Medicine. https://www.yalemedicine.org/news/covid-19-variants-of-concern-omicron.

47. Ashkelon: Traces of the Indian Coronavirus Variant Detected | Ministry of Health. https://www.gov.il/en/departments/news/24062021-03.

48. Recovery Protocol Update Due to the Omicron Variant | Ministry of Health. https://www.gov.il/en/departments/news/08122021-05.

49. Liu, Y. & Rocklöv, J. The effective reproductive number of the Omicron variant of SARS-CoV-2 is several times relative to Delta. *J Travel Med* **29**, (2022).

50. Lauer, S. A. *et al.* The Incubation Period of Coronavirus Disease 2019 (COVID-19) From Publicly Reported Confirmed Cases: Estimation and Application. *Ann Intern Med* (2020) doi:10.7326/M20-0504.

51. Linton, N. M. *et al.* Incubation Period and Other Epidemiological Characteristics of 2019 Novel Coronavirus Infections with Right Truncation: A Statistical Analysis of Publicly Available Case Data. *J Clin Med* **9**, 538 (2020).

52. He, X. *et al.* Temporal dynamics in viral shedding and transmissibility of COVID-19. *Nat Med* **26**, 672–675 (2020).

53. Gandhi, M., Yokoe, D. S. & Havlir, D. V. Asymptomatic Transmission, the Achilles’ Heel of Current Strategies to Control Covid-19. *New England Journal of Medicine* (2020) doi:10.1056/nejme2009758.

54. Sheehan, M. M., Reddy, A. J. & Rothberg, M. B. Reinfection Rates Among Patients Who Previously Tested Positive for Coronavirus Disease 2019: A Retrospective Cohort Study. *Clinical Infectious Diseases* (2021) doi:10.1093/CID/CIAB234.

55. Mossong, J. L. *et al.* Social contacts and mixing patterns relevant to the spread of infectious diseases. *PLoS Med* **5**, (2008).

56. Yechezkel, M. *et al.* Human mobility and poverty as key drivers of COVID-19 transmission and control. *BMC Public Health* **21**, 1–13 (2021).

57. Subjects - Live Births. https://www.cbs.gov.il/en/subjects/Pages/Live-Births.aspx.

58. Fertility Rate - the Haredi Institute for Public Affairs. https://machon.org.il/en/series/fertility-rate/.

59. COVID-19 Datasets- Government Data. https://data.gov.il/dataset/covid-19.
